# Supplementary material for: Are Shockley-Read-Hall and ABC models valid for lead halide perovskites?
Source: Nat Commun. 2021 Jun 7;12:3329. doi: 10.1038/s41467-021-23275-w (PMC8185072; doi:10.1038/s41467-021-23275-w)
Supplement: Supplementary file 1 — Supplementary Information [file 41467_2021_23275_MOESM1_ESM.pdf]

Supplementary Information.

Are Shockley-Read-Hall and ABC models valid for lead halide perovskites?

Alexander Kiligaridis,[1] Pavel A. Frantsuzov,[2] Aymen Yangui,[1] Sudipta Seth,[1] Jun Li,[1] Qingzhi An,[3] Yana Vaynzof[3] and Ivan G. Scheblykin[1]

[1]*Chemical Physics and NanoLund, Lund University, P.O. Box 118, 22100 Lund, Sweden*

[2]*Voevodsky Institute of Chemical Kinetics and Combustion, Siberian Brunch of the Russian Academy of Science, Institutskaya str. 3, 630090, Novosibirsk, Russia*

[3]*Integrated Center for Applied Physics and Photonic Materials (IAPP) and Centre for Advancing Electronics Dresden (cfaed), Technical University of Dresden, Dresden, Germany*

[*]**Corresponding Author:** *ivan.scheblykin@chemphys.lu.se*

Table of content:

Supplementary Note 1. Photoluminescence Microscopy Setup 2

Supplementary Note 2. Measurement of the PL(f,P) map 3

Supplementary Note 3. Measurements of the absolute PLQY 6

Supplementary Note 4. Monitoring and minimizing the effect of PL enhancement/bleaching during the PLQY(f,P) measurements 9

Supplementary Note 5. Detailed sample preparation procedure 11

Supplementary Note 6. SEM images, absorption, and PL spectra 13

Supplementary Note 7. PL decays in the single pulse and quasi-CW regimes 15

Supplementary Note 8. PLQY(W) plotted in the traditional way 16

Supplementary Note 9. Theoretical calculations and fitting 17

Supplementary Note 10. Photon emission and recycling in perovskite films 32

Supplementary Note 11. CW regimes for different models (data for Fig. 4 in the main text) 35

Supplementary Note 12. Fitting procedures for all models 36

Supplementary Note 13. Results of the fitting of the experimental data by ABC, SRH and SRH+ models 38

Supplementary references 41

Supplementary Note 1. Photoluminescence Microscopy Setup

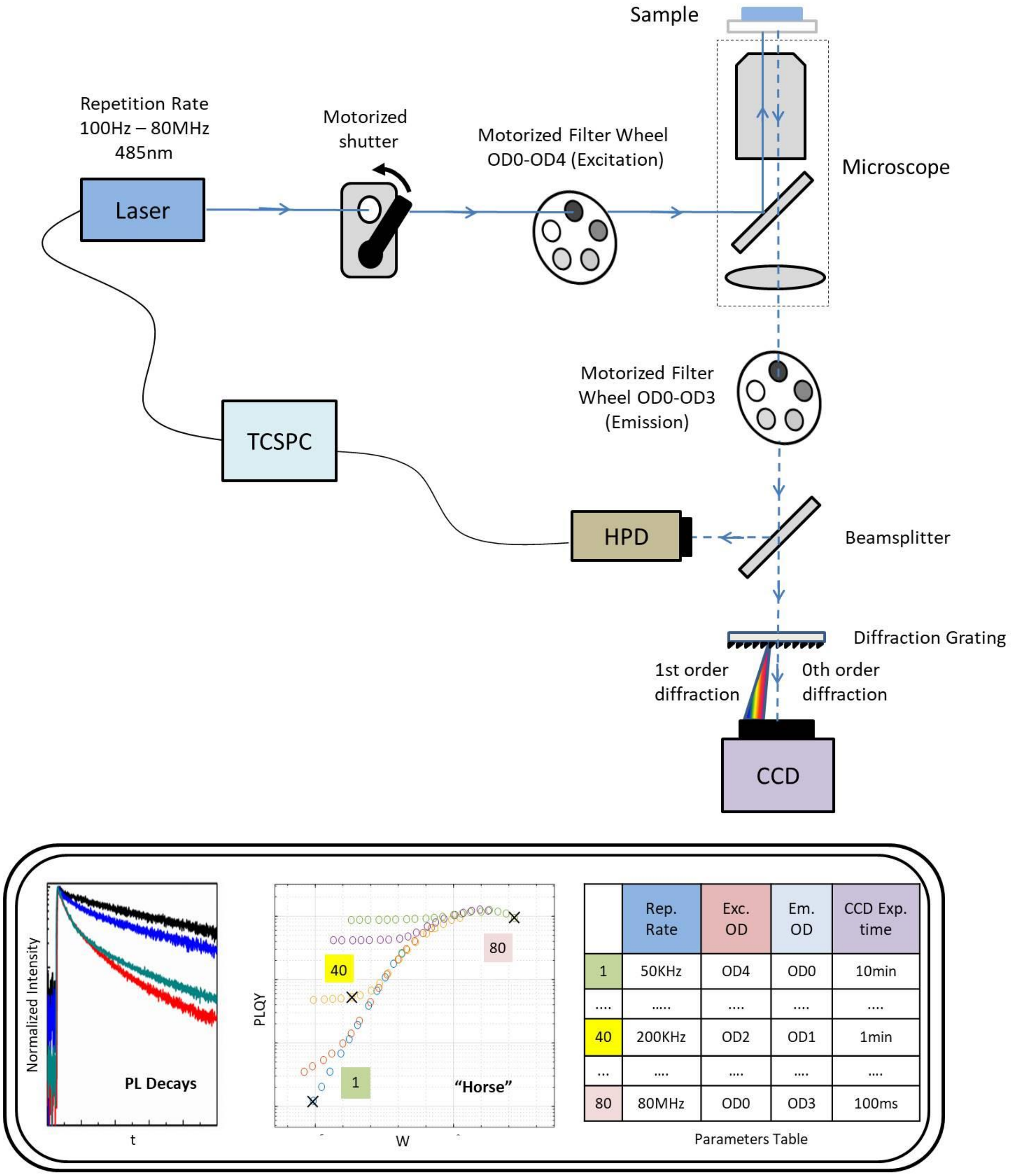

	Rep. Rate	Exc. OD	Em. OD	CCD Exp. time
1	50KHz	OD4	OD0	10min
....	.....	....	....	....
40	200KHz	OD2	OD1	1min
...	....	....	....	....
80	80MHz	OD0	OD3	100ms

Supplementary Figure 1.1. Schematic of the PL microscopy setup designed for measurements of PLQY(f,P) maps. The bottom panel shows the list of pre-defined parameters of the setup used by the automated control system to measure the 85 data points (numbered 1 to 85) of the PLQY(f,P) map. Points 1, 40 and 85 are highlighted in the exemplary PLQY(f,P) plot. PL decays are also measured for selected f and P combinations, by redirecting part of the emission with a beamsplitter to a hybrid photodetector (HPD) connected to a time correlated single photon counting (TCSPC) module. PL spectra were measured by adding a transmission diffraction grating in front of the CCD camera.

The PLQY(f,P) map and PL decays were measured in a custom-built photoluminescence microscopy setup (Supplementary Figure 1.1). A pulsed 485 nm laser (Pico Quant, 150 ps pulse width) was used to excite the sample through an objective lens (Olympus 40X, NA = 0.6) of a wide-field fluorescence microscope (Olympus IX-71). The emission of the sample was then collected by the same objective and captured by one of two detectors. The first detector is a EM CCD (Princeton Inst. ProEM 512B) for measuring PL(f,P)) (See Supplementary Note 3 on how PL(f,P) is converted to PLQY(f,P)). The second detector is a hybrid photomultiplier detector (HPD, Picoquant PMA Hybrid-42) which is connected to a time correlated single photon counting (TCSPC) module (Picoquant, Picoharp 300) for the measurement of PL decays kinetics. The instrumental response function minimum width is 200 ps.

Supplementary Note 2. Measurement of the PL(f,P) map

The PL(f,P) maps measured in this work consisted of 85 combinations of pulse fluences and laser frequencies. Five different pulse fluences were used, namely P1=4.1x10^8, P2=4.9x10^9, P3=5.1x10^{10}, P4=5.5x10^{11} and P5=4.9x10^{12} photons/cm^2, while the frequency varied between 100Hz and 80MHz.

In Supplementary Table 2.1 we present the five power fluences P1-P5 in alternative units of charge carrier density -n_0 (cm^{-3}), calculated using the following equation:

$$n_0 = \frac{P \cdot Absorptance}{d} \qquad (2.1)$$

where n_0 is the charge carrier density (cm^{-3}), P is the pulse fluence (photons/cm^2), d is the thickness of the perovskite layer (260 nm) and Absorptance is the fraction of the excitation light absorbed by the sample, which was estimated to 65% (the remainder light was mostly scattered and reflected).

Supplementary Table 2.1 Values of the five different pulse fluences P1-P5 (photons/cm^2) and their corresponding charge carrier densities n_{0_1} - n_{0_5} (cm^{-3}), estimated for sample thickness of 260 nm and 65% absorption.

Pulse Fluence P (photons/cm^2)	Charge Carrier Density n_0 (cm^{-3})
P1 = 4.1x10^8	n_{0_1} = 1.04x10^{13}
P2 = 4.9x10^9	n_{0_2} = 1.24x10^{14}
P3 = 5.1x10^{10}	n_{0_3} = 1.3x10^{15}
P4 = 5.5x10^{11}	n_{0_4} = 1.37x10^{16}
P5 = 4.9x10^{12}	n_{0_5} = 1.24x10^{17}

To measure a full PL(f,P) map, the CCD acquires a series of PL images, one for each combination of the laser repetition rate f and the pulse fluence P. This is achieved by utilizing a custom LabVIEW software that controls all parts of the setup. The software reads from an input table (Supplementary Table 2.2) each row containing a combination of parameters for each individual measurement: (i) repetition rate

of the laser (f), (ii) optical filter in the laser beam to regulate the pulse power fluence (Exc.OD), (iii) optical attenuation of the emission to protect the detector from saturation (Em.OD) and (iv) exposure time of the CCD (Exp.t). The pulse power fluence and the emission attenuation is controlled by two motorized filter wheels placed in the excitation and emission path, respectively, each containing a set of neutral optical density (OD) filters (Supplementary Figure 1.1).

$MAPbI_3$ films often exhibit photobleaching and photobrightening effects upon exposure to light. We paid great attention to minimizing and monitoring these effects by (i) keeping the samples under a nitrogen environment throughout all measurements, (ii) adding a motorized shutter, also automatically controlled by the software, blocking the excitation beam during the time necessary for the filter wheels to rotate between each acquisition, (iii) introducing a reference point (P1, 80MHz) which is measured multiple times during the acquisition of a full PL(f,P) map (Supplementary Note 4) and (iv) measuring additional PL(f,P) maps on the same spot on the sample.

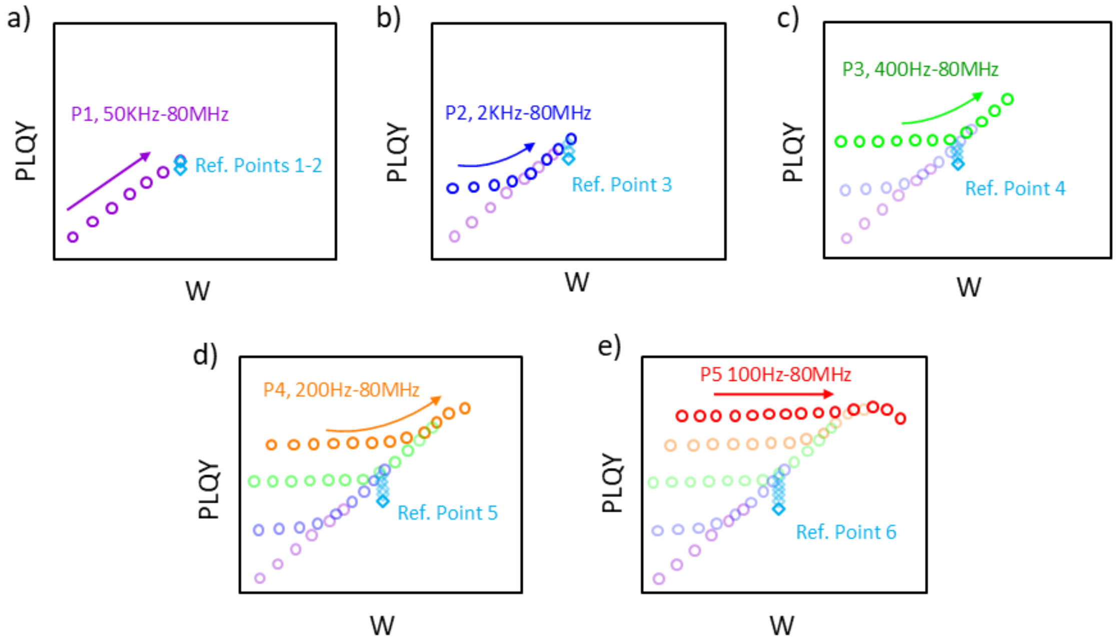

Supplementary Figure 2.1. The sequence in which one complete PL(f,P) map is measured. The entire scan is performed in a sequence of 5 measurements as schematically shown: a) P=P1, f scanned, with the reference points 1 and 2 measured before and after the scan respectively; b) P=P2, f scanned followed by the reference point 3; c) P=P3, f scanned followed by the reference point 4; d) P=P4, f scanned followed by the reference point 5; e) P=P5, f scanned and the full round is finished by measuring the final reference point 6. The change in the reference points in the figure is exaggerated to make it clearly distinguishable.

Supplementary Table 2.2. Parameters for acquisition of the PL(P,f) maps for the $MAPbI_3$ samples studied in this work. The rows marked in red indicate the reference point, which was repeatedly measured to track any changes in the sample's PL due to photoinduced bleaching/brightening.

#	P	f (kHz)	Em. OD	Exp. t (s)
1	1	80000	0	0.1
2	1	50	0	400
3	1	100	0	400
4	1	200	0	200
5	1	500	0	200
6	1	1000	0	100
7	1	2000	0	50
8	1	5000	0	10
9	1	10000	0	10
10	1	20000	0	10
11	1	40000	0	1
12	1	80000	0	0.1
13	2	2	0	800
14	2	4	0	800
15	2	10	0	400
16	2	20	0	400
17	2	50	0	200
18	2	100	0	200
19	2	200	0	200
20	2	500	0	200
21	2	1000	0	20
22	2	2000	0	10
23	2	5000	0	2
24	2	10000	0	1
25	2	20000	0	0.5
26	2	40000	0	0.1
27	2	80000	0	0.1
28	1	80000	0	0.1
29	3	0.4	0	400
30	3	1	0	400
31	3	2	0	200
32	3	4	0	100
33	3	10	0	20
34	3	20	0	20
35	3	50	0	20
36	3	100	0	5
37	3	200	0	5
38	3	500	0	1
39	3	1000	0	0.1
40	3	2000	0	0.1
41	3	5000	0	0.1
42	3	10000	1	0.1
43	3	20000	1	0.1
44	3	40000	2	0.1
45	3	80000	2	0.1
46	1	80000	0	0.1
47	4	0.2	0	80
48	4	0.4	0	40
49	4	1	0	10
50	4	2	0	10
51	4	4	0	5
52	4	10	0	1
53	4	20	0	0.5
54	4	50	0	0.5
55	4	100	0	0.1
56	4	200	0	0.1
57	4	500	0	0.1
58	4	1000	1	0.1
59	4	2000	1	0.1
60	4	5000	2	0.1
61	4	10000	2	0.1
62	4	20000	3	0.1
63	4	40000	3	0.1
64	4	80000	3	0.1
65	1	80000	0	0.1
66	5	0.1	0	10
67	5	0.2	0	5
68	5	0.4	0	2
69	5	1	0	1
70	5	2	0	1
71	5	4	0	0.5
72	5	10	0	0.1
73	5	20	0	0.1
74	5	50	1	0.1
75	5	100	1	0.1
76	5	200	1	0.1
77	5	500	2	0.1
78	5	1000	2	0.1
79	5	2000	3	0.1
80	5	5000	3	0.1
81	5	10000	4	0.1
82	5	20000	4	0.1
83	5	40000	4	0.1
84	5	80000	4	0.1
85	1	80000	0	0.1

Supplementary Note 3. Measurements of the absolute PLQY

Measurements of the absolute PLQY using an integrating sphere

By definition, external PLQY is the ratio of the number of photons emitted to the total number of photons absorbed:

$$\mathrm{PLQY} = \frac{\#\, photons\ emitted}{\#\, photons\ absorbed} \tag{3.1}$$

The number of photons absorbed and emitted by the samples under excitation with the same laser source as used for PLQY(f,P) mapping were obtained using an integrating sphere (Horiba ,Quanta-ϕ) coupled through a fiber to a compact CCD spectrometer (Thorlabs CCS200/M). The measurements of the laser intensity (spectrum) with and without the sample as well as the sample PL gives:

$$\#\, photons\ absorbed \; = \; \alpha \left(\int_{-\infty}^{+\infty} c(\lambda)\, Exc_{blank}(\lambda) d\lambda \; - \; \int_{-\infty}^{+\infty} c(\lambda)\, Exc_{sample}(\lambda)\, d\lambda \right) \tag{3.2}$$

$$\#\, photons\ emitted = \alpha \int_{-\infty}^{+\infty} c(\lambda)\, PL(\lambda)\, d\lambda \tag{3.3}$$

where $Exc_{blank}(\lambda)$, $Exc_{sample}(\lambda)$ are the measured spectra of the laser without and with the sample in the sphere, respectively; $PL(\lambda)$ is the spectrum of the sample emission, $\alpha c(\lambda)$ is the total light collection coefficient for the wavelength λ and $c(\lambda)$ presents the spectral sensitivity of the integrating sphere, fiber and spectrometer combined. It is important that all spectra in Eqs. (3.2) and (3.3) are presented in the units of photons/nm.

Spectral sensitivity calibration of the PLQY measurement with integrating sphere

To calibrate the spectral sensitivity of the setup, we used the spectrometer (via the sphere and the fiber) to measure the light of a Tungsten Halogen calibration lamp (OceanOptics LS-1-CAL.). Then, $c(\lambda)$ was obtained by dividing the known lamp spectrum with the one measured.

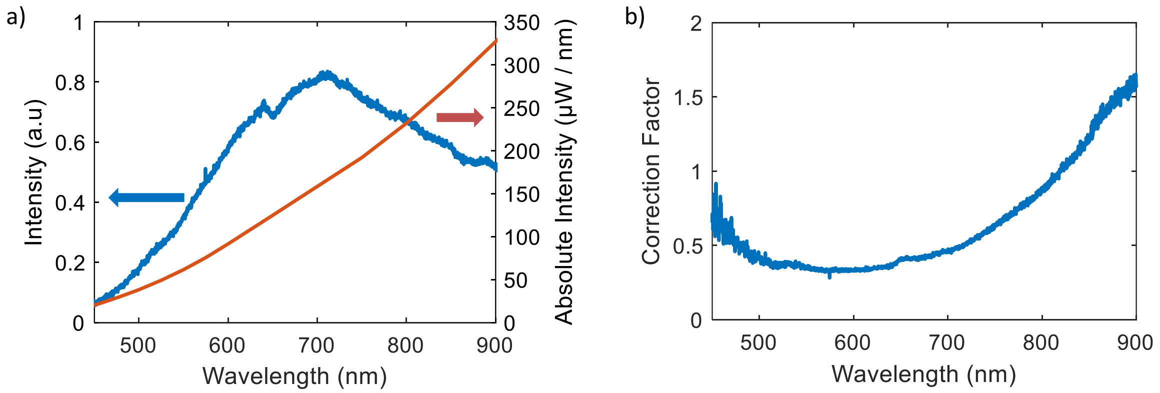

Supplementary Figure 3.1. (a) Spectrum of the calibration lamp measured by a spectrometer when guided through the sphere-fiber (blue) and the calibration spectrum of the lamp (orange) calibrated by standards that provide traceability to the National Institute of Standards and Technology (NIST). Note that the intensity units of the calibration spectrum are μW/nm, the units of the measured spectrum (intensity) are also power/nm. (b) Correction curve is obtained by dividing the calibration spectrum with the one obtained from the spectrometer.

Since the standard lamp spectrum is given in the units of power/nm, we multiply all measured spectra by λ to compensate for the relation between energy and number of photons (Eqs. (3.4)-(3.6)).

$$Number\ of\ Photons(\lambda) = \frac{Energy(\lambda)}{Energy\ per\ photon(\lambda)} \tag{3.4}$$

$$Energy\ per\ photon(\lambda) = \frac{hc}{\lambda} \tag{3.5}$$

$$Number\ of\ Photons(\lambda) = \frac{\lambda}{constant} Energy(\lambda) \tag{3.6}$$

Thus, the final calibrated spectra $Exc'_{blank}(\lambda)$, $Exc'_{sample}(\lambda)$ and $PL'(\lambda)$ in units of photons/nm are obtained as:

$$Exc'_{blank}(\lambda)\left(\frac{photons}{nm}\right) = \lambda\, c(\lambda)\, Exc_{blank}(\lambda) \tag{3.7}$$

$$Exc'_{sample}(\lambda)\left(\frac{photons}{nm}\right) = \lambda\, c(\lambda)\, Exc_{sample}(\lambda) \tag{3.8}$$

$$PL'(\lambda)\left(\frac{photons}{nm}\right) = \lambda\, c(\lambda)\, PL(\lambda) \tag{3.9}$$

Conversion of the PL intensity measured by the microscope to the absolute PLQY measured by the integrating sphere

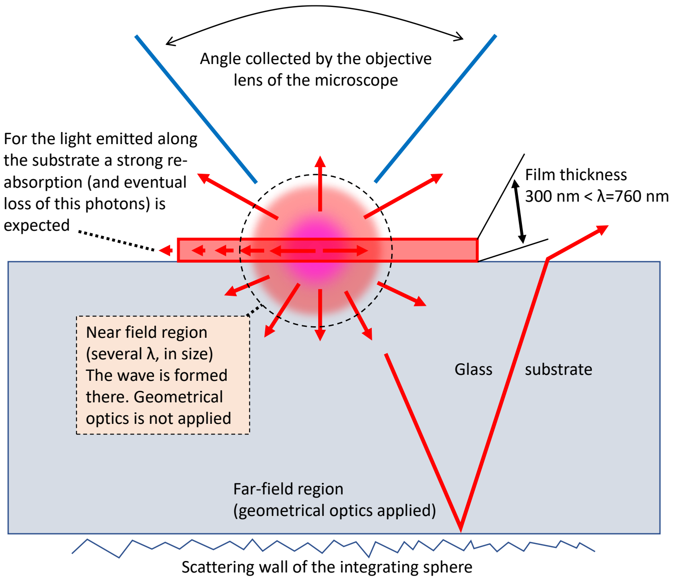

Supplementary Figure 3.2. Propagation of PL light emitted by a thin film with a thickness smaller than the wavelength. Collection of PL by a fluorescence microscope and by an integrating sphere. The escape probability of the light emitted along the film is very low because of re-absorption. Since PLQY <<1, a very small fraction of photons originally emitted along the film will be able to leave the film from its edges.

The integrating sphere collects all light which was able to escape. The objective lens collects only the light within the collection angle. There is a ratio between the light collected by the objective and by

the detector sitting in the integrating sphere. Below, it will be described how we measured this coefficient.

Using the setup described in the Supplementary Note 1, we first measured the evolution of the PL intensity as a function of the laser repetition rate and pulse fluence (PL(f,P) map). To convert PL intensity to absolute PLQY, we have developed the procedure described below.

We have the following equations:

$$\frac{\#\, emitted\ photons}{time} = a_{PL}\,\frac{PL}{t_{exp}} \qquad (3.10)$$

$$\frac{\#\, absorbed\ photons}{time} = \beta\,\frac{W}{\frac{hc}{\lambda_{exc}}} \qquad (3.11)$$

$$PLQY = \frac{\#\, emitted\ photons}{\#\, absorbed\ photons} = \frac{a_{PL}\frac{PL}{t_{exp}}}{\frac{\lambda_{exc}}{hc}W\beta} = \left(\frac{a_{PL}\ hc}{\lambda_{exc}\beta}\right)\cdot\frac{PL}{Wt_{exp}} = b\,\frac{PL}{Wt_{exp}} \qquad (3.12)$$

where a_{PL} is the total light detection efficiency of the micro-PL setup for a given sample. It is determined by the CCD quantum efficiency, light collection geometry of the microscope objective, the emission angular diagram of the sample and the transmission coefficient of all optical components; PL is the number of measured counts by the CCD over an exposure time of t_{exp}, β is the fraction of the laser light absorbed by the sample, λ_{exc} is the excitation wavelength and W is the excitation power in Watts.

In equations 3.12, constant b combines all these factors together. By knowing the value of b, we can convert the measured PL intensity to an absolute PLQY.

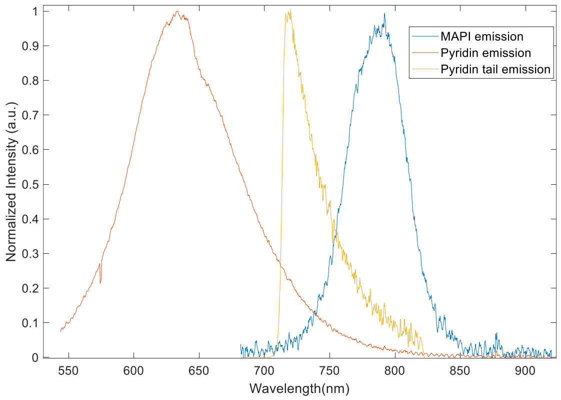

Supplementary Figure 3.3. Normalized spectra of a Pyridine 2 dye (red), Pyridine 2 dye low energy tail measured using a 695 nm long-pass filter to detect only the low energy tail of the emission spectrum (yellow). PL spectrum of $MAPbI_3$ (blue) is shown for comparison.

The simplest approach of estimating b is by measuring the PL intensity and the absolute PLQY of a calibration sample. Since b is dependent on the sample properties, the calibration sample should have similar optical properties to the $MAPbI_3$ films under investigation. We selected two types of calibration samples: first, the $MAPbI_3$ film itself and second, a Pyridine 2 laser grade dye (Lambda FYSIK) dispersed in a thin (ca 100 - 200 nm) PMMA film. Pyridine 2 has strong absorption at our excitation wavelength and a broad PL extending to the near infrared region. The absorption measured in the integrating

sphere was 64.6% for $MAPbI_3$ and 63.7% for the Pyridine 2 sample at an excitation wavelength of 485 nm. PLQY of the Pyridine sample was 12%. Because Pyridine 2 has a broad PL band, we placed a long-pass filter in front of the CCD detector to measure only the low energy tail emission which is spectrally close to the emission band of $MAPbI_3$ (Supplementary Figure 3.3). This filtered emission was used to calculate the b coefficient. Supplementary Table 3.1 shows the b values obtained using the dye calibration sample, compared to those obtained using the $MAPbI_3$ film.

Supplementary Table 3.1 Obtained b values from Pyridine 2 and $MAPbI_3$ calibration samples. Each value was obtained from a different spot of the respective samples, which reveals certain inhomogeneities in the films.

Reference Sample	b	$\langle b \rangle \pm \sigma_b$
Pyridin 2 in PMMA	0.0413	0.0450±0.0038
	0.0419	
	0.0458	
	0.0509	
	0.0449	
$MAPbI_3$ film	0.0544	0.0460±0.0087
	0.0370	
	0.0467	

We observe larger fluctuations in b for the $MAPbI_3$ samples, due to following factors:

i) $MAPbI_3$ films exhibit worse spatial PL homogeneity compared to the spin cast dye. Even though the excitation spot size for the PLQY measurement using the integrating sphere is 14 mm^2 and gives an averaged PLQY over almost the whole sample, the PL microscope spot is probing only a much smaller 900 μm^2 area within the film and is thus more sensitive to any local PL inhomogeneities.

ii) $MAPbI_3$ film is less stable under light illumination and exposure to air/humidity.

iii) PLQY of $MAPbI_3$ is strongly dependent on the excitation power density. Any mismatch of excitation power densities in the two measurements (PL and PLQY) manifests as error in estimation of b. The PLQY of the dye on the other hand shows no dependence on excitation power density.

Supplementary Note 4. Monitoring and minimizing the effect of PL enhancement/bleaching during the PLQY(f,P) measurements.

For each sample the PL intensity at the reference point (P1, 80MHz) was measured multiple times during the acquisition of a complete PL(P,f) map (see Supplementary Note 2, Supplementary Figure 2.1) to assess PL bleaching/brightening effects which may influence PLQY and PL decay dynamics. The results of these control measurements for each sample are presented in Supplementary Figure 4.1.

Considering that we noticed that the maximum changes occur at low pulse fluence, we selected the reference point at P1 in order to maximize the apparent effect of enhancement/bleaching. As can be seen in Supplementary Figure 4.1, the maximum change of the PLQY always below a factor of two for the entire experiment. This should be noted when assessing the relative accuracy of the entire measurement. Note also that the different samples showed different response to light which was also dependent on the power density. PL enhancement was dominant for all samples except of G/P/MAPI/P film which showed very slight bleaching.

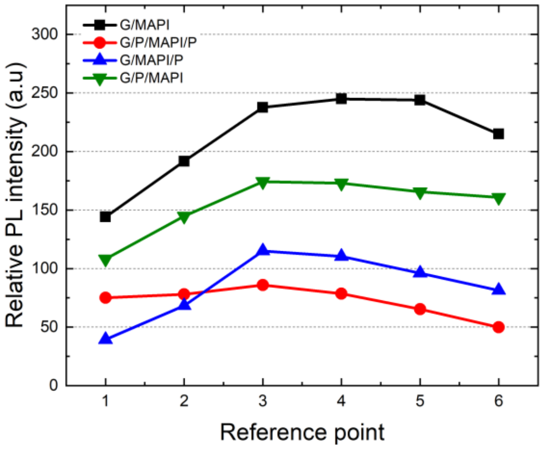

Supplementary Figure 4.1. Changes of PL measured at the reference point (P1, 80MHz) during the scanning over the f,P space. All samples show similar trends, which is a PL enhancement during the lower excitation power density (P1-P2) scans and a PL bleaching at the higher ones (P4-P5).

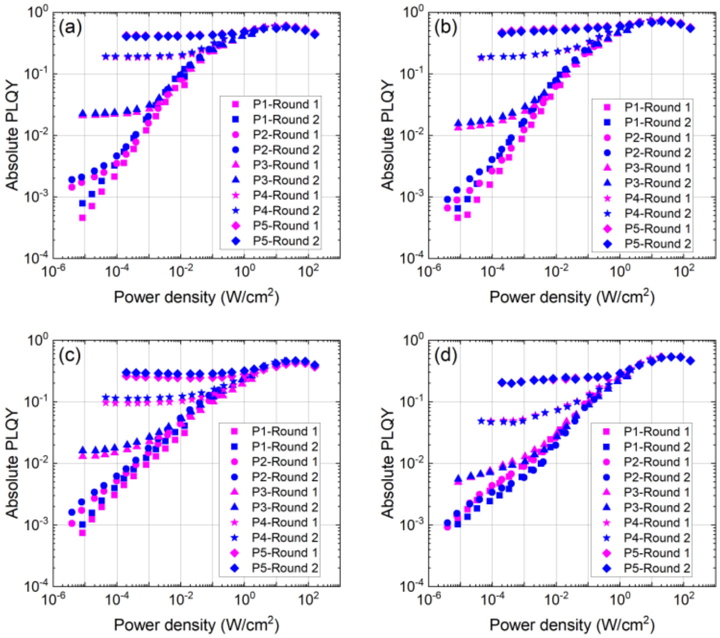

Supplementary Figure 4.2. Same spot/region -two rounds measurements of the PLQY maps for (a) G/MAPI, (b) G/PMMA/MAPI, (c) G/MAPI/PMMA, and (d) G/PMMA/MAPI/PMMA.

For each sample, the same spot was measured twice acquiring two complete PL(P,f) maps to further track any effect of PL bleaching/brightening or other degradation effects (Supplementary Figure 4.2). In general, such effects were only apparent in the low power density regime, and were mostly negligible in the middle and high power excitation regimes. In Supplementary Figure 4.3, we also present the measured PLQY(f,P) maps for two different spots/regions of the G/MAPI sample, which shows that the sample exhibits analogous results for different regions.

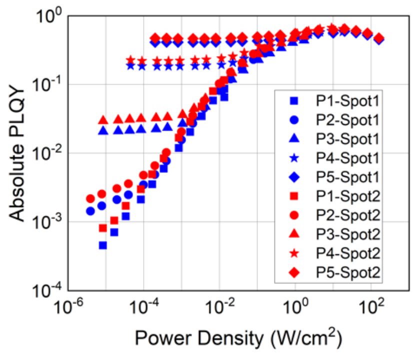

Supplementary Figure 4.3. Measurements of the PLQY maps for G/MAPI sample on two different regions of the same film.

Supplementary Note 5. Detailed sample preparation procedure

Materials: Methylammonium iodide (CH_3NH_3I), lead acetate trihydrate ($Pb(CH_3CO_2)_2 3H2O$, >99.0%) and PMMA (Mn 212000) were purchased from Great Cell Solar, Tokyo Chemical Industry and Polymer Source Inc, respectively. Hypophosphorous acid (HPA, 50wt% in water), chlorobenzene (anhydrous, 99.8%) and N,N-Dimethylformamide (DMF, anhydrous 99.8%) were purchased from Sigma-Aldrich, using as received.

Solution preparation: Perovskite precursor was prepared according to the previous work,[1,2] in detail 40 %wt perovskite solution was prepared with 1:3 molar ratio of lead acetate trihydrate and methylammonium iodide dissolving in dimethylformamide. Additionally, 8‰ (HPA/DMF volume ratio) amount of HPA were added into the perovskite precursor. PMMA solution was prepared by dissolving 10 mg PMMA in 1 ml chlorobenzene.

Interface Combinations: The $MAPbI_3$ films were prepared with different combinations of interfaces of glass substrate (G), PMMA (P) and $MAPbI_3$ (MAPI); 1) G/MAPI, 2) G/MAPI/P 3) G/P/MAPI and 4) G/P/MAPI/P (Supplementary Figure 5.1).

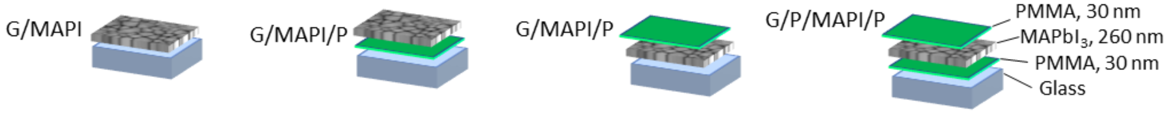

Supplementary Figure 5.1 Illustration of the interface combinations of the four different samples.

Thin films fabrication: Glass substrates (microscope cover slips, 0.17 mm thickness) were ultrasonically cleaned with 2 % hellmanex detergent, deionized water, acetone, and isopropanol, followed by 10 min oxygen plasma treatment. The cleaned substrates were then transferred into a drybox (RH < 1 %) for further manipulations. For samples with PMMA layer beneath the perovskite film, PMMA was spin-coated on the clean substrates with 3000 rpm for 30 s and annealed at 100 °C for 10 min. The perovskite precursor was spin-coated at 2000 rpm for 60 s on glass or glass/PMMA substrates, following by a 25 s dry air blowing, a 5 min room temperature drying and a 10 min 100°C annealing. For the samples with PMMA on top, no further annealing was applied after depositing PMMA (3000 rpm for 30 s) on the top of the perovskite layer.

XRD measurements were conducted on samples prepared on glass and measured at room temperature in ambient on a Rigaku SmartLab diffractometer equipped with a 9 kW rotating copper anode. A 2D HyPix3000 detector in a coupled θ − 2θ scan (beam collimator 0.2 mmφ) at a detector distance of 110 mm was used to gather 2D-XRD data. The 1D-profile was obtained after integrating a 46° wide central wedge of the 2D-diffraction pattern, correction of the background and removal of contributions of Kα2 using the Rigaku 2DP software.

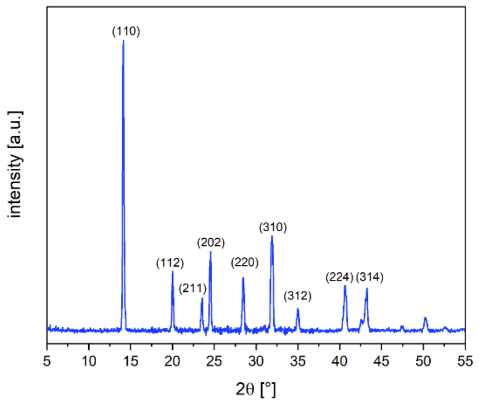

Supplementary Figure 5.2 X-ray diffraction (XRD) measurement of the glass/MAPI sample.

Solar cell preparation. The MAPbI3 films prepared by the methodology described above were used to prepare photovoltaic devices, details are given below.

Pre-patterned indium tin oxide (ITO) coated glass substrates (PsiOTech Ltd., 15 Ohm/sqr) were ultrasonically cleaned with 2 % hellmanex detergent, deionized water, acetone, and isopropanol, followed by 8 min oxygen plasma treatment. In a drybox (RH<3%), PTAA (1.5 mg/ml dissolved in toluene) was spin-coated on the substrates at 4000 rpm for 30 s and annealed at 100 C for 10 minutes. The perovskite active layer was deposited as described in the film preparation section. Next, the samples were transferred into a nitrogen filled glove box, where PCBM (20 mg/ml dissolved in chlorobenzene) was dynamically spin-coated at 2000 rpm 30 s on the perovskite layer followed by a 10 min annealing at 100 C. Finally, BCP (0.5 mg/ml dissolved in isopropanol) was spin-coated at 4000 rpm for 30 s, following by 80 nm thermally evaporated silver.

Solar cell characterisation. The current density-voltage (J-V) curve was measured using a computer controlled Keithley 2450 Source Measure Unit under simulated AM 1.5 sunlight with 100 mW/cm^2 irradiation (Abet Sun 3000 Class AAA solar simulator). The light intensity was calibrated with a Si

reference cell (NIST traceable, VLSI) and corrected by measuring the spectral mismatch between the solar spectrum, the spectral response of the perovskite solar cell and the reference cell. The cells were scanned from forward bias to short-circuit and reverse at a rate of 0.2 V/s by employing a mask to eliminate the overestimation of the photocurrent. The photovoltaic performance parameters are: V_{oc} = 1.128 V, J_{sc} = -22.27 mA/cm^2, FF = 80.63 %, PCE = 20.25 % (Supplementary Figure 5.3).

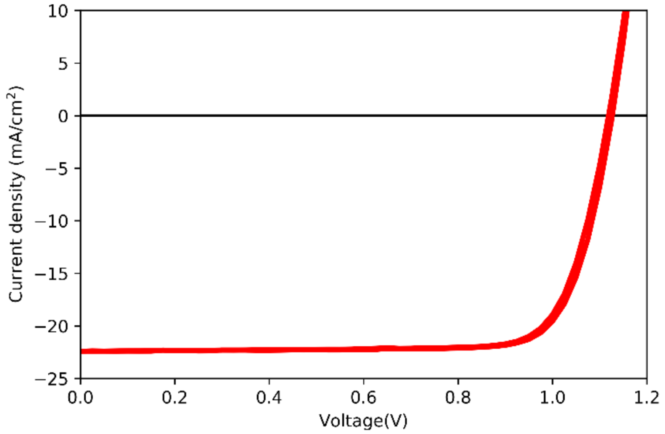

Supplementary Figure 5.3 Photovoltaic performance of the MAPbI3 material studies in this work.

Supplementary Note 6. SEM images, absorption, and PL spectra

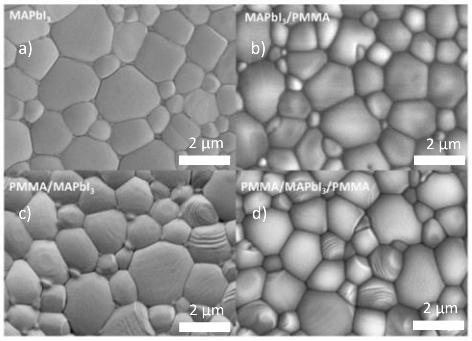

Supplementary Figure 6.1. SEM images of the four different samples studied: a) glass/MAPI, b) glass/MAPI/PMMA, c) glass/PMMA/MAPI and d) glass/PMMA/MAPI/PMMA. Gemini 500 (ZEISS, Oberkochen, Germany) was used to acquire the images at an acceleration voltage of 1 kV using a secondary electron detector.

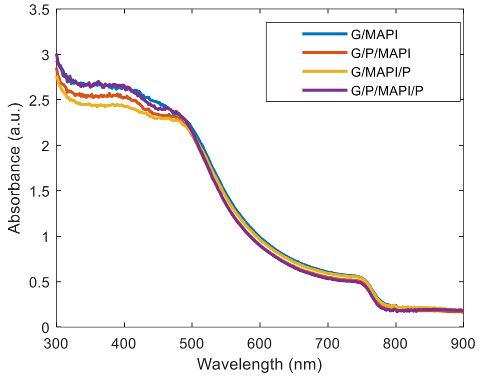

Supplementary Figure 6.2. Absorption spectra for the four different samples; G/MAPI (blue), G/P/MAPI (red), G/MAPI/P (yellow), G/P/MAPI/P (purple). The spectra are very similar. The measurements were carried out using the ordinary measurement scheme (direct transmission), that is why the apparent optical density in the blue region (OD=2.5) is higher than expected for 260 nm think films (OD≈1.8) due to wavelength-dependent light scattering.[3]

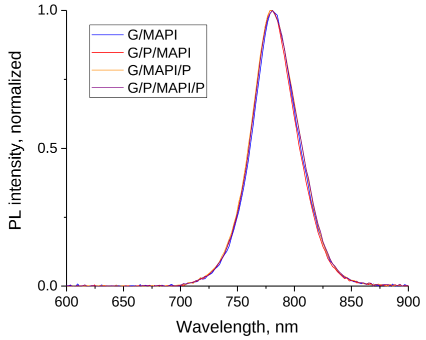

Supplementary Figure 6.3. Photoluminescence spectra were measured using home-built photoluminescence microscopy setup (Supplementary Note 1), by limiting the image by a slit and using a transmission diffraction grating in the front of the CCD camera, spectral resolution was 5 nm. We used 485 nm pulsed laser (80 MHz, 0.16 W / cm^2 excitation power density) for excitation.

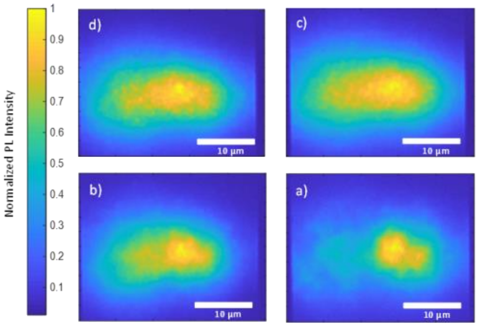

Supplementary Figure 6.4. PL images of (a) G/MAPI samples, (b) G/P/MAPI, (c) G/MAPI/P, (d) G/P/MAPI/P. The images show the size and shape of the laser excitation spot as well as a slight spatial inhomogeneity of PL due to the grainy structure of the films.

Supplementary Note 7. PL decays in the single pulse and quasi-CW regimes

For each sample, PL decays were measured for different pulse fluences (from P1 to P5) and at different laser frequencies ranging from 30 kHz to 10 MHz. The purpose of these measurements was to track the evolution of the PL decays during the transition from a single pulse to a quasi-CW regime. In particular, for P4 pulse fluence, at 30 kHz all samples are in the single pulse regime and at 1MHz, all of them are at the quasi-CW regime. As is shown in Supplementary Figure 7.1, there appears to be no significant difference in the PL decays between these two frequencies, rendering the examination of PL decays on their own incapable of distinguishing between the two different excitations regimes. It is interesting that for $MAPbI_3$ without PMMA coverage increasing of the repetition rate leads to a faster PL decay, while for the samples with PMMA coverage the behavior is the opposite. See Kudriashova *et. al.*[4] where a similar behavior was observed for other types of surface treatments.

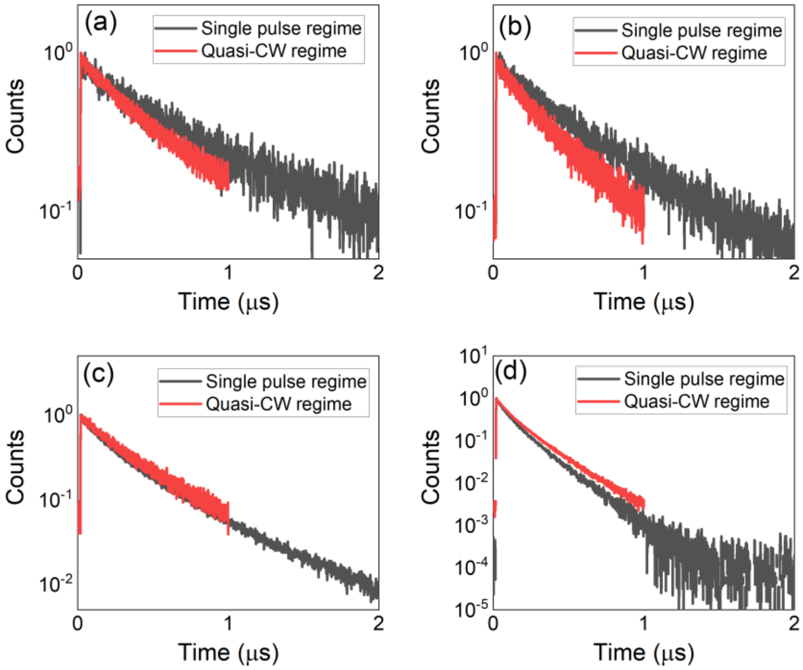

Supplementary Figure 7.1. PL decays of (a) G/MAPI, (b) G-P/MAPI, (c) G/MAPI/P, and (d) G/P/MAPI/P, measured at pulse fluence P4 on the single pulse (black, 30 kHz) and the quasi-CW (red, 1 MHz) excitation regimes.

Supplementary Note 8. PLQY(W) plotted in the traditional way

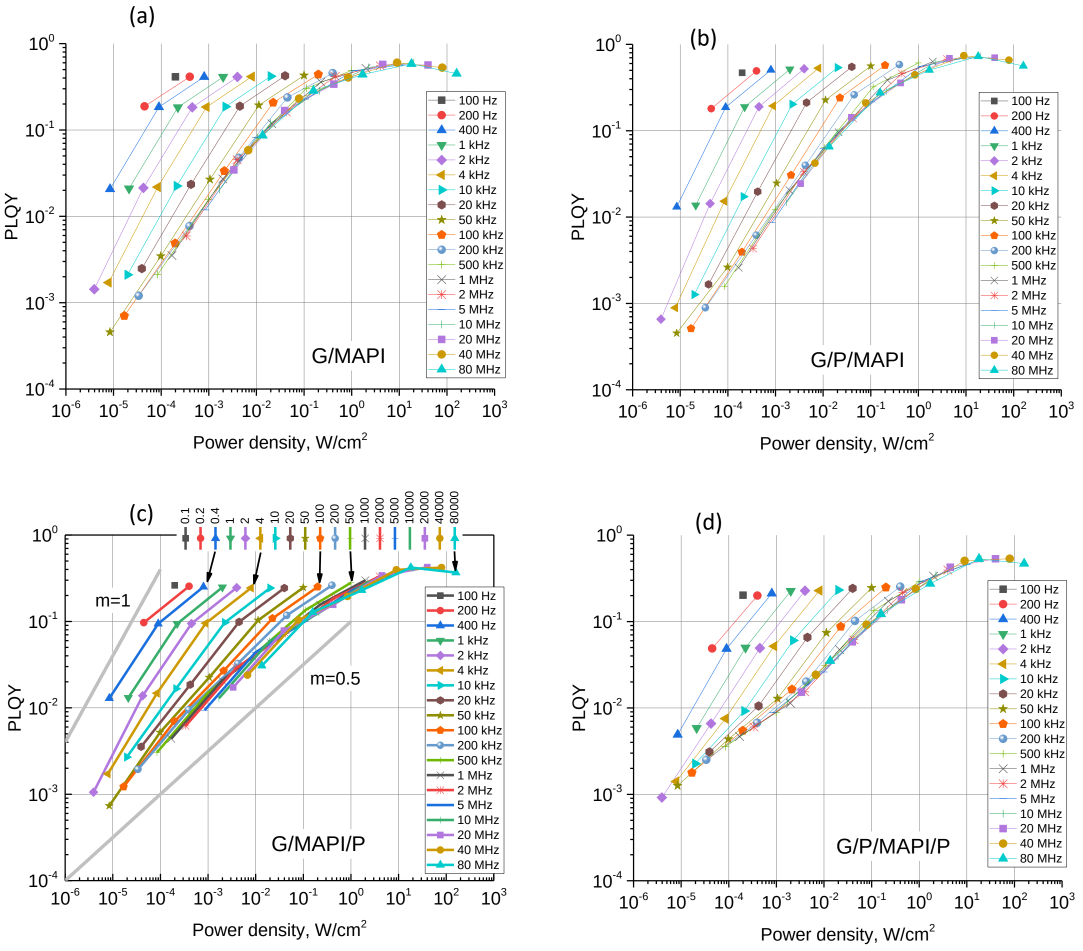

Supplementary Figure 8.1. PLQY(f,P) data plotted in the traditional way: data points measured at the same repetition rate are connected by lines. There 19 different frequencies and 5 pulse fluences (P1,P2,P3,P4 and P5) used. (a) – sample G/MAPI, (b) – G/P/MAPI, (c) – G/MAPI/P, (d) – G/P/MAPI/P.

Supplementary Note 9. Theoretical calculations and fitting

9.1 Notes about diffusion and photon recycling

PLQY calculated in this section is the so-called external PLQY because we operate with the experimentally determined PL and excitation intensities. Photon recycling is included indirectly in the theoretical models, because the rate constants of radiative recombination and Auger trapping can be seen as re-normalized constants corresponding to the light/energy propagation conditions for the particular sample, see Supplementary Note 10 for details.

We do not explicitly include charge diffusion in the model. This is rationalized by the fact that charge carrier diffusion in $MAPbI_3$ is fast, with diffusion coefficients on the order of 1 cm^2/s reported in literature.[5,6] Thus, assuming 1D diffusion toward the surface, initially inhomogeneous (exponentially distributed due to the excitation light attenuation in the film) charge distribution homogenizes over the 260 nm thickness of the film with the characteristic time $t=\langle x^2\rangle/2D$, $t=(260\times10^{-7}\ cm)^2/(2\times1\ cm^2/s) = 3.4\ 10^{-10}s = 0.34$ ns. In our measurements and calculations, we are interested in the dynamics at timescales longer than 10 ns, that is why we can assume equilibrated homogeneous distribution of charge carriers over the entire thickness of the film.

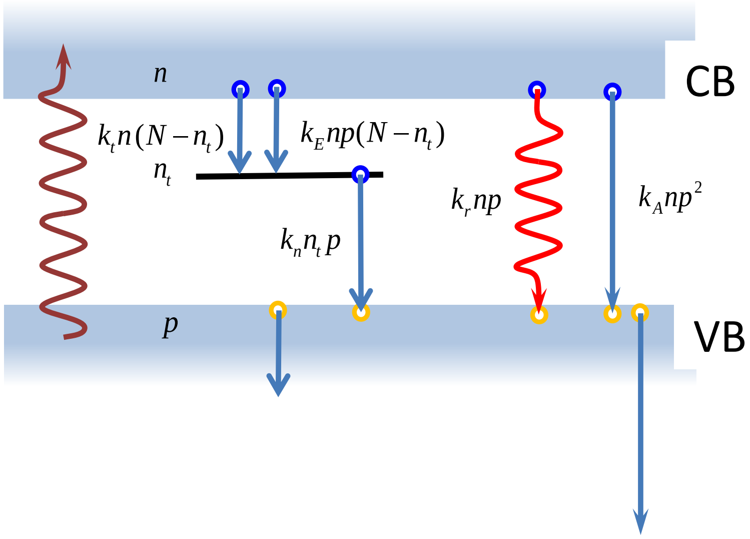

Supplementary Figure 9.1. Theoretical SRH+ model (the extended SRH model)

9.2. List of designations related to the excitation conditions:

f [s^{-1}]– laser pulse repetition rate;

$h\nu$ [J] - photon energy;

P [photons/cm^2] – laser pulse fluence, number of photons hitting the unit area in one pulse;

n_0 [cm^{-3}]- density of charge carriers generated by one laser pulse, $n_0 = Absorptance \cdot P/d$, where d is the thickness of the sample (see Supplementary Note 2);

W [W/cm^2] – average excitation power density, for pulsed excitation: $W = f \cdot P \cdot h\upsilon$.

G [cm^{-3} s^{-1})] - average density of generated charge carriers per second, $G = W \cdot \frac{Absorptance}{d\, h\upsilon}$.

9.3 Formulation of the SRH+ model

Kinetic equations of the model:

$$\frac{d}{dt}n(t) = G(t) - k_r np - k_t(N - n_t)n - k_E np(N - n_t) - k_A np^2 \tag{9.1}$$

$$\frac{d}{dt}n_t(t) = k_t(N - n_t)n + k_E np(N - n_t) - k_n n_t p \tag{9.2}$$

$$\frac{d}{dt}p(t) = G(t) - k_r np - k_n n_t p - k_A np^2 \tag{9.3}$$

Where $G(t)$ is density of the photogenerated carriers per second [$\mathrm{cm}^{-3}s^{-1}$], $n(t)$,$n_t(t)$ and $p(t)$ are the densities of electrons in the CB, trapped electrons and holes respectively, N is the density of traps. k_r,k_t, k_n,k_E, and k_A are radiative electron-hole recombination rate constant, electron trapping rate constant, non-radiative recombination rate constant of a trapped electron and a free hole, Auger assisted electron trapping rate constant, and Auger assisted electron-hole recombination rate constant, respectively.
The PL intensity within the model is given by the expression:

$$PL(t) = k_r n(t)p(t) \tag{9.4}$$

Shockley – Reed – Hall model follows from SRH+ model by setting k_E and k_A to zero.

9.4 The ABC model

Derivation of the ABC model from the SRH+ model

In the limit of $k_n \to \infty$ the density of the trapped electrons n_t equals to 0 as follows from Eq.(9.2). Thus $n(t) = p(t)$, which is the condition for the ABC model.

From Eq. (9.1) we get

$$\frac{d}{dt}n(t) = G(t) - k_r n^2 - k_t Nn - k_E Nn^2 - k_A n^3.$$

Or in another form

$$\frac{d}{dt}n(t) = G(t) - An - Bn^2 - Cn^3, \tag{9.5}$$

where $A = k_t N$, $B = k_r + k_E N$, and $C = k_A$.
PL intensity can be found as

$$PL(t) = k_r n^2(t) \tag{9.6}$$

Pulse excitation experiment in the ABC model

In order to find PL intensity in the pulse excitation regime the following equation has to be solved:

$$\frac{d}{dt} n(t) = -An - Bn^2 - Cn^3 \tag{9.7}$$

with the periodic boundary condition (periodic solution):

$$n(0) = n(T) + n_0$$

where n_0 is the carrier density generated by one pulse, T is the time period between pulses.
The PLQY can be found as:

$$PLQY = \frac{k_r}{n_0} \int_0^T n^2(t) dt \tag{9.8}$$

Note that here PLQY is external PLQY.

Quasi-CW regime of the ABC model

As seen from the periodic boundary condition, the variation of the $n(t)$ value during the period between the laser pulses is equal to n_0. Thus, if the condition

$$n(t) \gg n_0$$

satisfies, we can set $n(t)$ equal to its averaged value, which means that the system is in quasi-CW regime

$$n(t) = \tilde{n}$$

Substituting this expression to the right-hand side of Eq. (9.7) we get:

$$\frac{d}{dt} n(t) = -A\tilde{n} - B\tilde{n}^2 - C\tilde{n}^3 \tag{9.9}$$

Integrating Eq. (9.9) with respect to time from 0 to T we obtain:

$$-n_0 = -A\tilde{n}T - B\tilde{n}^2 T - C\tilde{n}^3 T$$

Introducing the averaged density of the photogenerated carriers per second $G = \frac{n_0}{T}$, we get the following equation:

$$G = A\tilde{n} + B\tilde{n}^2 + C\tilde{n}^3 \tag{9.10}$$

Solving Eq. (9.10) at a given value G we can get $\tilde{n}$ and then can calculate the PL quantum yield:

$$PLQY = \frac{k_r\tilde{n}^2}{G} \tag{9.11}$$

PL kinetics in the ABC model in the low excitation limit.

If the following inequalities apply

$$Bn \ll A \text{ and } Cn \ll B$$

Eq. (9.7) can be rewritten as

$$\frac{d}{dt}n(t) = -An \tag{9.12}$$

Periodic solution of Eq. (9.12) is

$$n(t) = \frac{n_0 \exp(-At)}{1 - \exp(-AT)} \tag{9.13}$$

In **quasi-CW** regime $AT \ll 1$ and $n(t)$ is equal to its averaged value:

$$\tilde{n} = \frac{n_0}{AT} = \frac{G}{A} \tag{9.14}$$

Substituting to Eq. (9.11) we have

$$PLQY = \frac{k_r}{A^2}G \tag{9.15}$$

In the **single pulse** regime $AT \gg 1$ and the density of electrons is

$$n(t) = n_0 \exp(-At)$$

Substituting to Eq. (9.6) we have

$$PL(\,t) = k_r n_0^2 \exp(-2At) \tag{9.16}$$

Using Eq. (9.8) we got

$$PLQY = \frac{k_r}{2A} n_0$$
(9.17)

9.5 The SRH+ model

Pulse excitation experiment in the SRH+ model

In order to extract the PL intensity in the pulse excitation regime the following set of equations has to be solved:

$$\frac{d}{dt} n(t) = -k_r np - k_t(N - n_t)n - k_E np(N - n_t) - k_A np^2 \quad (9.18)$$
$$\frac{d}{dt} n_t(t) = k_t(N - n_t)n + k_E np(N - n_t) - k_n n_t p \quad (9.19)$$
$$\frac{d}{dt} p(t) = -k_r np - k_n n_t p - k_A np^2 \quad (9.20)$$

with the periodic boundary conditions (periodic solution):

$$n(0) = n(T) + n_0$$
$$p(0) = p(T) + n_0$$
$$n_t(0) = n_t(T)$$

And the condition of charge conservation:

$$n(t) + n_t(t) = p(t) \quad (9.21)$$

The PLQY can be found as:

$$PLQY = \frac{k_r}{n_0} \int_0^T n(t)p(t)dt$$
(9.22)

Quasi-CW regime of the SRH+ model

As it is seen from the periodic boundary conditions, the variations of the values of $n(t)$, $p(t)$ and $n_t(t)$ are not larger than n_0 during the period between the laser pulses. However, in order for the system to be in a quasi-CW regime, it is not required for all these values to be much greater than n_0. As it is seen from Figure 3a the PL intensity could decay for more than an order of magnitude during the time period T within a quasi-CW regime. It can be explained by a fast decay of the electron density

$n(t)$ according to Eq. (9.18). Thus, the quasi-CW regime conditions for the SRH+ model can be written in the following form:

$$p(t) \gg n_0 \quad \text{and} \quad n_t(t) \gg n_0$$

The densities $p(t)$ and $n_t(t)$ can be considered as equal to their averaged values due to the presence of photodoping

$$p(t) = \tilde{p} \quad \text{and} \quad n_t(t) = \tilde{n}_t$$

Substituting these expressions to the right-hand sides of Eqs. (9.18-9.20) we get:

$$\frac{d}{dt}n(t) = -k_r n(t)\tilde{p} - k_t(N - \tilde{n}_t)n(t) - k_E n(t)\tilde{p}(N - \tilde{n}_t) - k_A n(t)\tilde{p}^2 \tag{9.23}$$
$$\frac{d}{dt}n_t(t) = k_t(N - \tilde{n}_t)n(t) + k_E n(t)\tilde{p}(N - \tilde{n}_t) - k_n \tilde{n}_t \tilde{p} \tag{9.24}$$
$$\frac{d}{dt}p(t) = -k_r n(t)\tilde{p} - k_n \tilde{n}_t \tilde{p} - k_A n(t)\tilde{p}^2 \tag{9.25}$$

Integrating Eqs. (9.23-9.25) with respect to time from 0 to *T* we obtain:

$$-n_0 = -k_r \bar{n}\tilde{p}T - k_t(N - \tilde{n}_t)\bar{n}T - k_E \bar{n}\tilde{p}(N - \tilde{n}_t)T - k_A \bar{n}\tilde{p}^2 T \tag{9.26}$$
$$0 = k_t(N - \tilde{n}_t)\bar{n}T + k_E \bar{n}\tilde{p}(N - \tilde{n}_t)T - k_n \tilde{n}_t \tilde{p}T \tag{9.27}$$
$$-n_0 = -k_r \bar{n}\tilde{p}T - k_n \tilde{n}_t \tilde{p}T - k_A \bar{n}\tilde{p}^2 T \tag{9.28}$$

where $\bar{n}$ is the averaged density of electrons:

$$\bar{n} = \frac{1}{T}\int_0^T n(t)dt$$

Eq. (9.22) gives the expression for the PL quantum yield

$$PLQY = \frac{k_r}{n_0}\bar{n}\tilde{p}T$$

Equations (9.26-9.28) are not independent. We can obtain an additional equation by averaging Eq. (9.21)

$$\bar{n} + \tilde{n}_t = \tilde{p}$$

Finally, we obtain the following system of equations:

$$G - k_r \bar{n}\tilde{p} - k_n \tilde{n}_t \tilde{p} - k_A \bar{n}\tilde{p}^2 = 0 \tag{9.29}$$
$$k_t(N - \tilde{n}_t)\bar{n} + k_E \bar{n}\tilde{p}(N - \tilde{n}_t) - k_n \tilde{n}_t \tilde{p} = 0 \tag{9.30}$$

$$\bar{n} + \tilde{n}_t = \tilde{p} \tag{9.31}$$

Solving them at a given value of G we can extract $\bar{n}$, $\tilde{n}_t$ and $\tilde{p}$, and then able to calculate the PL quantum yield:

$$PLQY = \frac{k_r \bar{n} \tilde{p}}{G} \tag{9.32}$$

From Eqs. (9.30) and (9.31) we obtain

$$k_t(N - \tilde{n}_t)\bar{n} + k_E \bar{n}(\bar{n} + \tilde{n}_t)(N - \tilde{n}_t) - k_n \tilde{n}_t(\bar{n} + \tilde{n}_t) = 0 \tag{9.33}$$

From Eq. (9.33) the variable $\tilde{n}_t$ can be expressed as a function of $\bar{n}$:

$$\tilde{n}_t = \xi(\bar{n})$$

where

$$\xi(\bar{n}) = \frac{\sqrt{\bar{n}^2(k_t + k_E\bar{n} + k_n - k_E N)^2 + 4\bar{n}N(k_n + k_E\bar{n})(k_t + k_E\bar{n})} - \bar{n}(k_t + k_E\bar{n} + k_n - k_E N)}{2(k_n + k_E\bar{n})} \tag{9.34}$$

Thus, the system of equations (9.29-9.31) is equivalent to the following equation:

$$F(\bar{n}) = G \tag{9.35}$$

Where the function $F(\bar{n})$ is given by the following expression:

$$F(\bar{n}) = k_r\bar{n}(\bar{n} + \xi(\bar{n})) + k_n\xi(\bar{n})(\bar{n} + \xi(\bar{n})) + k_A\bar{n}(\bar{n} + \xi(\bar{n}))^2 \tag{9.36}$$

Solving Eq. (9.35) one can obtain $\bar{n}$ for given value G in quasi-CW regime. The $\tilde{n}_t$ and $\tilde{p}$ values are

$$\tilde{n}_t = \xi(\bar{n}) \qquad \tilde{p} = \xi(\bar{n}) + \bar{n}$$

The PL quantum yield can be expressed in this regime using Eq. (9.32). Periodic solution of the Eq. (9.23) has a form:

$$n(t) = \frac{n_0 \exp(-\gamma t)}{1 - \exp(-\gamma T)} \tag{9.37}$$

where

$$\gamma = k_r\tilde{p} + k_t(N - \tilde{n}_t) + k_E\tilde{p}(N - \tilde{n}_t) + k_A\tilde{p}^2$$

PL intensity (9.4) in quasi-CW regime

$$PL(t) = \frac{k_r n_0 \tilde{p}}{1 - \exp(-\gamma T)} \exp(-\gamma t)$$

As these equations shows, that PL during the period T between the laser pulses can decay to any level which is determined on the total decay rate γ, see the cartoon in Fig. 1 d,e,f in the main text.

9.6 SRH model

Kinetic equations of the SRH model can be obtained from Eqs**.** (9.12-9.20) by setting $k_A = 0$ and $k_E = 0$:

$$\frac{d}{dt}n(t) = -k_r np - k_t(N - n_t)n \quad (9.38)$$

$$\frac{d}{dt}n_t(t) = k_t(N - n_t)n - k_n n_t p \quad (9.39)$$

$$\frac{d}{dt}p(t) = -k_r np - k_n n_t p \quad (9.40)$$

These equations can be considered as low excitation approximation of SRH+ model when the following inequalities apply (negligible rates of Auger-assisted processes):

$$k_A p^2 \ll k_t N \text{ and } k_E p \ll k_t$$

Low excitation intensity in the SRH model when PLQY is low and there is no trap filling

Let's consider the SRH model when additional conditions are applied:

1) PLQY is low (radiative rate<<non-radiative radiative)

$$k_r p \ll k_t N \quad (9.41)$$

2) There is no trap filling, which means that

$$n_t \ll N, \quad (9.42)$$

In general, conditions (9.41) and (9.42) correspond to low excitation conditions, however, before we solve the equations, we cannot write the condition for the generation rate *G* explicitly.

In this limit the equations (9.38-9.40) can be simplified:

$$\frac{d}{dt}n(t) = -k_t Nn \quad (9.43)$$

$$\frac{d}{dt}n_t(t) = k_t Nn - k_n n_t p \quad (9.44)$$

$$\frac{d}{dt}p(t) = -k_n n_t p \qquad (9.45)$$

Periodic solution of Eq. (9.43) is

$$n(t) = \frac{n_0 \exp(-k_t N t)}{1-\exp(-k_t N T)} \qquad (9.46)$$

Single pulse regime in SRH model (low PLQY, no trap filling)

When the following conditions applied:

$$k_t NT \gg 1 \text{ and } k_n n_0 T \gg 1$$

the system is in single pulse regime. It follows from Eq. (9.46) that

$$n(t) = n_0 \exp(-k_t N t)$$

meaning that the electronic density disappears on the timescale of $t_d \sim (k_t N)^{-1}$.

It can be seen from Eq. (9.43) that the density of holes is not changing significantly within this time period (t_d) , if

$$t_d k_n n_0 = \frac{k_n n_0}{k_t N} \ll 1$$

In this case, we can substitute $p(t)$ in Eq. (9.4) by its initial value:

$$p(t) = n_0$$

Thus we get:

$$PL(t) = k_r n_0^2 \exp(-k_t N t) \qquad (9.47)$$

Using Eq. (9.16) we obtain

$$PLQY = \frac{k_r}{k_t N} n_0 \qquad (9.48)$$

Quasi-CW regime in the SRH model (low PLQY, no trap filling)

In quasi-CW regime the densities $p(t)$ and $n_t(t)$ must be much larger than n_0 and can be considered as equal to their averaged values

$$p(t) = \tilde{p} \quad \text{and} \quad n_t(t) = \tilde{n}_t$$

By integrating Eqs. (9.43-9.45) over time we get

$$G = k_n \tilde{n}_t \tilde{p} \tag{9.49}$$
$$G = k_t N \bar{n} \tag{9.50}$$
$$\bar{n} + \tilde{n}_t = \tilde{p} \tag{9.51}$$

Because $G = \frac{n_0}{T}$, Eq. (9.49) can be presented as

$$n_0 = k_n \tilde{n}_t \tilde{p} T$$

while $\tilde{n}_t, \tilde{p} \gg n_0$ the following condition must apply to bring the system to quasi-CW regime:

$$k_n n_0 T \ll 1$$

From Eq. (9.50) we get:

$$\bar{n} = \frac{G}{k_t N} \tag{9.52}$$

Substituting Eq. (9.52) and (9.41) to Eq. (9.49) we get the following equation:

$$G = k_n \tilde{p} \left(\tilde{p} - \frac{G}{k_t N} \right)$$

Solution of this equation gives us average concentration of holes:

$$\tilde{p} = \frac{1}{2} \left[\sqrt{\left(\frac{G}{k_t N} \right)^2 + \frac{4G}{k_n}} + \frac{G}{k_t N} \right] \tag{9.53}$$

and from Eq. (9.51) we obtain average concentration of trapped electrons:

$$\tilde{n}_t = \frac{1}{2} \left[\sqrt{\left(\frac{G}{k_t N} \right)^2 + \frac{4G}{k_n}} - \frac{G}{k_t N} \right] \tag{9.54}$$

Square root dependence of PLQY in the SRH model (low PLQY, no trap filling)

If to the existing conditions of low PLQY and absence of trap filling (conditions (9.41)) and (9.42)) we add the following conditions for the generation rate:

$$G \ll G_r$$

where

$$G_r = \frac{k_t^2 N^2}{k_n} \tag{9.55}$$

Then from Eqs. (9.53-9.54) we obtain

$$\tilde{p} = \tilde{n}_t = \sqrt{\frac{n_0}{k_n T}} = \sqrt{\frac{G}{k_n}} \tag{9.56}$$

Then Eq. (9.32) and (9.52) lead to the square root PLQY dependence on excitation intensity:

$$PLQY = \frac{k_r}{k_t N \sqrt{k_n}} G^{1/2} \tag{9.57}$$

Substituting Eq. (9.56) and Eq. (9.44) to Eq. (9.9) results in mono exponential PL kinetics in this case:

$$PL(t) = \frac{k_r n_0^{3/2}}{\sqrt{k_n T}(1 - \exp(-k_t N T))} \exp(-k_t N t) \tag{9.58}$$

Explicit conditions for generation rate *G* and their consequences

After solving the equations of the SRH model we can write explicitly the conditions (9.41) and (9.42). The first condition (9.41) is that PLQY is low is equivalent to:

$$G \ll G_s$$

where

$$G_s = \frac{k_t^2 N^2 k_n}{k_r^2} \tag{9.59}$$

The second condition (9.42) is absence of trap filling condition, which can be presented in the form:

$$G \ll G_f$$

where

$$G_f = N^2 k_n \tag{9.60}$$

Thus, to observe the square root PLQY dependence on the excitation power density which was just discussed above the generation rate G must be smaller than G_r and G_s and G_f:

$$G \ll G_r \text{ and } G \ll G_f \text{ and } G \ll G_s$$

In general behavior of the PLQY(W) upon creasing the power density depends on which of the values G_r , G_s or G_f is the smallest, or, in other words, which of the conditions breaks first upon increasing of the generation rate (which of the limiting values G_r , G_s or G_f is the smallest).

Standard square rood dependence with saturation

In the simplest case (straight line in the quasi-CW regime) the smallest is G_S. This is also equivalent to $k_r \geq k_n, k_n$
In this case the square root dependence turns to the saturation with $PLQY{\sim}1$ at

$$G \gg G_s$$

See the curve labeled SRH in Figure 4 c in the main text.

Reaching an ABC-like behavior at higher excitation in the SRH model

Let's consider the case, when G_r is the smallest of G_r, G_s and G_r. This is equivalent to:

$$k_n \gg k_r, k_t$$

At the excitation power

$$G \gg G_r \quad (9.61)$$

And the other conditions still valid $G \gg G_{S,}\ G_f$

the density of holes Eq. (9.53) and trapped electrons Eq. (9.54) are

$$\tilde{p} = \frac{G}{k_t N}$$

$$\tilde{n}_t = \frac{k_t}{k_n} N$$

So, the quantum yield is

$$PLQY = \frac{k_r}{k_t^2 N^2} G \quad (9.62)$$

Eq. (9.62) is equivalent to the PLQY expression in the ABC model Eq. (9.17). It means that under the condition (9.61) the system is in the ABC-like regime.

See the series of curves labeled "k_n increases" in Figure 4 d in the main text which shows how the square-root dependence changes to the linear dependence (ABC – like behavior).

We can use these considerations to estimate the k_n value which is required to make ABC model valid for the excitation power density larger than $W_{\min} = 4 \times 10^{-6}$ W/cm^2 (the smallest value used in our experiments) and for 1 Sun power density $W_{\mathrm{sun}} = 0.1$ W/cm^2. The corresponding G values are:

$$G_{\min} = W_{\min} \cdot \frac{Absorptance}{d\, h\upsilon} \approx 2.5 \times 10^{17} \mathrm{cm}^{-3}\mathrm{s}^{-1}$$

$$G_{\mathrm{sun}} \approx 6.2 \times 10^{21} \mathrm{cm}^{-3}\mathrm{s}^{-1}$$

Using the parameter $k_t N = 5 \times 10^6 \mathrm{s}^{-1}$ found for G/P/MAPI/P sample we obtain for the first case

$$k_n \gg \frac{k_t^2 N^2}{G_{\min}} \approx 10^{-4} \mathrm{cm}^3\mathrm{s}^{-1}$$

and for the for the second case (1 Sun power):

$$k_n \gg 4 \times 10^{-9} \mathrm{cm}^3\mathrm{s}^{-1}$$

Reaching trap filling at high excitation in the SRH model

The third case is if G_f is the smallest of G_r, G_s and G_r. It happens if

$$k_t \gg k_n, k_r$$

In this case the system reaches trap filling at

$$G \sim G_f \tag{9.63}$$

Trap saturation manifests itself as changing PLQY(W) from the square root dependence to a faster growing around W corresponding to G_f as shown by the curve labeled "SRH Trap filling" in Figure 4 c in the main text.

9.7 Chemical doping and its influence on PLQY in quasi-CW regime

Let us assume that there is chemical doping in the system. Doping can be n-doping (extra electrons) or p-doping (extra holes).

Thus, the charge conservation condition in the system of Eq. (9.48-9.50) has to be substituted by:

$$\tilde{n} + \tilde{n}_t + c_d = \tilde{p}$$

where c_d is a density of additional charges due to chemical doping. Density c_d is positive for p-doped sample and negative for n-doped sample.

$$G - k_n \tilde{n}_t \tilde{p} = 0 \tag{9.64}$$

$$k_t N \tilde{n} - k_n \tilde{n}_t \tilde{p} = 0 \tag{9.65}$$

$$\tilde{n} + \tilde{n}_t + c_d = \tilde{p} \tag{9.66}$$

The system is equivalent to the following equation:

$$G = k_n \tilde{p} \left(\tilde{p} - c_d - \frac{G}{k_t N} \right) \tag{9.67}$$

Solving Eq. (9.65) we obtain

$$\tilde{p} = \frac{1}{2} \left[\sqrt{\left(c_d + \frac{G}{k_t N} \right)^2 + \frac{4G}{k_n}} + \left(c_d + \frac{G}{k_t N} \right) \right] \tag{9.68}$$

At very low excitation power

$$G \ll k_n c_d^2 , G \ll k_t N |c_d|$$

The solution of Eq. (9.68) in the case of positive c_d (p-doped sample) is

$$\tilde{p} = n_d$$

Thus, the quantum yield Eq. (9.32) is independent on G:

$$PLQY = \frac{k_r c_d}{k_t N}$$

If c_d is negative (n-doped samples) Eq. (9.68) gives

$$\tilde{p} = \frac{G}{k_n |c_d|}$$

And, consequently, the quantum yield Eq. (9.32) has a linear dependence on G:

$$PLQY = \frac{k_r G}{k_n |c_d| k_t N}$$

(9.69)

At higher excitation power

$$\frac{k_t^2 N^2}{k_n} \gg G \gg k_n n_d^2$$

Eq. (9.68) becomes equivalent to the result without doping Eq. (9.56), and quantum yield has the square root dependence (9.57) on excitation intensity.

The calculations above show that the presence of the chemical doping (both p-type or n-type) should result in strong change of the *PLQY(W)* dependence upon decreasing of the excitation power lower than a certain value which depends on the doping concentration, which is

$$G \ll k_n c_d^2$$

Since there is no such turnover observed in our experiments, it allows making an estimation of the maximal doping density in our samples:

$$c_d \ll \sqrt{\frac{G_{\min}}{k_n}}$$

Substituting parameter $k_n = 5.93 \times 10^{-12}\mathrm{cm}^3\mathrm{s}^{-1}$ and $G_{\min} = 2.5 \times 10^{17}\mathrm{cm}^{-3}\mathrm{s}^{-1}$ we get

$$c_d \ll 2 \times 10^{14}\mathrm{cm}^{-3}$$

9.8 Dependence on density of traps N, conditions for trap filling

Eqs. (9.29-9.31) for SRH model ($k_A = 0$ and $k_E = 0$):

$$G - k_r \tilde{n}\tilde{p} - k_n \tilde{n}_t \tilde{p} = 0 \quad (9.70)$$
$$k_t (N - \tilde{n}_t)\tilde{n} - k_n \tilde{n}_t \tilde{p} = 0 \quad (9.71)$$
$$\tilde{n} + \tilde{n}_t = \tilde{p} \quad (9.72)$$

Introducing unitless variables (densities of charges relative to the density of traps)

$$\hat{n} = \tilde{n}/N; \quad \hat{n}_t = \tilde{n}_t/N; \quad \hat{p} = \tilde{p}/N$$

Eqs. (9.68-9.70) can be rewritten as:

$$\hat{G} = k_r \hat{n}\hat{p} + k_n \hat{n}_t \hat{p} \quad (9.73)$$

$$k_t(1-\hat{n}_t)\hat{n} = k_n\hat{n}_t\hat{p} \quad (9.74)$$
$$\hat{n} + \hat{n}_t = \hat{p} \quad (9.75)$$

where $\hat{G} = G/N^2$ - renormalized averaged density of photogenerated charges.

The PLQY is provided by following formula:

$$PLQY = \frac{k_r\hat{n}\hat{p}}{\hat{G}} \quad (9.76)$$

Obviously, the solution of Eqs. (9.73 - 9.75) depends on parameters k_t, k_r, k_n, and $\frac{G}{N^2}$.

That means that the qualitative behavior of the PLQY in the quasi-CW regime as a function of the renormalized excitation fluence $\hat{G} = G/N^2$depends only on the values k_t, k_r, k_n , while the trap concentration N is a scaling parameter. In other words, the shape of the *PLQY(W)* depends only on the rate constants, while changing of N moves the dependence (or the whole PLQY(f,P) map) along the excitation power axis.

We are going to present a detailed analysis of the possible *PLQY(W)* regimes in a forthcoming theoretical work.

Supplementary Note 10. Photon emission and recycling in perovskite films

Photon reabsorption in thick (>>λ) films

Photon reabsorption/recycling is considered as an important process influencing the charge dynamics.[7] In our experimental study we compare samples of very similar geometries ensuring the effects of photon reabsorption/recycling to be similar, such that they cannot serve as the reasons for the differences between PLQY(f,P) maps and PL decay kinetics. Moreover, since the thickness of the studied $MAPbI_3$ films is only 260 nm, which is smaller than PL wavelength (760 nm), strictly speaking it is not possible to talk about photon reflection from film surfaces in terms of geometrical optics. Instead emission of a spatially-limited nano-scale media, the standard problem of nano-optics,[8] should be solved using explicit electrodynamic calculations. As we discuss below in detail, all influences on the charge carrier dynamics related to "photon recycling" in broad terms (both far field (photon reabsorption) and near field (energy transfer) effects), are included in our SRH+ model via "renormalized" radiative rate constant and the Auger trapping rate, respectively.

Photon re-absorption is a process which is widely discussed in relation to the photo-physics of MHP films and single crystals. It is generally assumed that a photon created inside a material (for example, a film), then propagates as a wave and reaches an interface with another dielectric media (*e.g.* air or

glass). At this interface, the wave partially or fully (total internal reflection) reflects toward the bulk of the crystal. In this way the wave becomes partially trapped inside the film and photons get re-absorbed by the semiconductor again because of the long propagation length in the material. Note here, that all these considerations are based on the geometrical optics which is valid only if the size of the crystal (thickness of the film) is substantially larger than the wavelength of light λ.

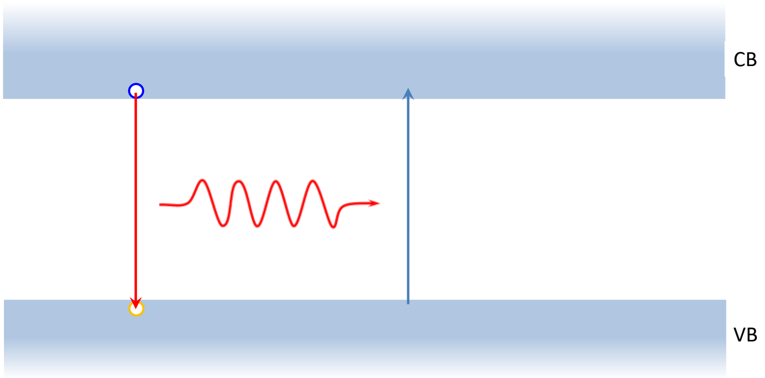

Supplementary Figure 10.1. Photon re-absorption in a semiconductor when a photon generates a free electron and hole. This process is considered in the SRH+ model due to effective renormalization of the radiative recombination rate constant k_r (it is lower due to re-absorption).

Let us consider a film with thickness >> λ. If the emitted photon generated by recombination of an e-h pair is reabsorbed, it generates a new e-h pair (Supplementary Figure 10.1). Thus, density of the free electrons and holes remains unchanged. Photon reabsorption can be considered in the SRH model by replacing the internal radiative recombination rate constant k_r^{int} by its renormalized value which is lower than the k_r value of the case in which the reabsorption was absent:

$$k_r = (1 - \xi)k_r^{int} \tag{10.1}$$

where ξ is the probability of generation of a free electron and a hole by photon reabsorption. So, in the limit of the applicability of the geometrical optics, photon reabsorption decreases the effective radiative recombination rate. Because it needs to compete with NR recombination (unchanged), decreasing of k_r due to re-absorption decreases PLQY of the sample.

Parasitic absorption without formation any charge carriers or optical excitation of an electron directly to the trap states are not considered due to very low absorption coefficients these processes in comparison with the absorption above the bandgap for our sampled prepared on glass.

<u>Thin (<λ) perovskite thin films: energy transfer *vs* photon reabsorption</u>

Consideration of processes such as the emission and absorption of photons inside an object with a size of the order of the wavelength seems to be very problematic. The formation of an electromagnetic field (photon) occurs in the near-field region, the size of which is several times larger than the wavelength. This means that one cannot say that a photon is emitted from a "point" inside the perovskite film of ~260 nm thickness (the thickness of our samples and a typical thickness for solar

cells) and then the wave travels until the interface. In other words, the bulk of the perovskite film and its interfaces cannot be treated independently, instead, the problem of the emission of the whole layer must be solved explicitly using quantum electrodynamics. The solution of the problem will lead to a certain constant of radiative recombination which then has to be employed in the modelling of charge dynamics. Note that we in principle do not consider an "external" and "internal" PLQY herein. PLQY is inherent to the particular sample geometry and, if the characteristic dimensions of the sample are smaller than the wavelength, cannot be split into "internal" and "external".
However, there is a process that occurs at the nanoscale (near field) which results in similar effects to those of photon reabsorption in the far-field - the Förster resonance energy transfer (FRET) (schematically shown in Supplementary Figure 10.2).

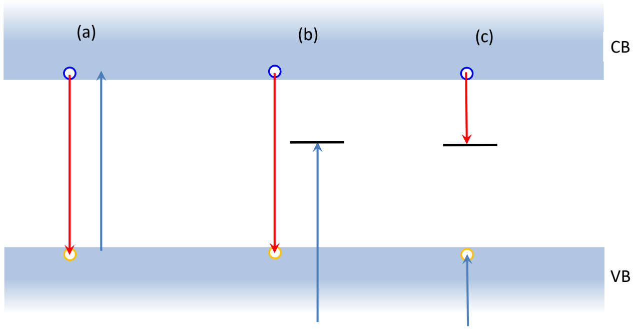

Supplementary Figure 10.2. Energy transfer processes in a semiconductor: a) free e^- and h^+ disappear, new e^- and h^+ appear in a different location; b) free e^- and h^+ disappear, new e^- from the VB is excited to the trap in a new location; c) free e^- goes to the trap in a new location, and free h^+ disappears by filling by an electron jumping up from a deeper VB level; Red arrow – e^- goes down loosing energy, e^- goes up gaining energy.

As is shown in Supplementary Figure 10.2a, the energy released from recombination of an e-h pair is transferred over a few nanometer distance and creates another e-h pair. Obviously, this process does not affect the carrier density and does not change the radiative recombination rate and hence the PLQY. This process is "invisible" in the SRH model and can influence the charge dynamics only indirectly *via* increasing the overall charge diffusion. However, the energy can be transferred to a trap state directly as shown in Supplementary Figure 10.2b and c. The energy of a recombining e-h pair is used to excite an electron from the valence band to a trap. One can view this process as an Auger assisted trapping,[9] because it involves three particles: free electron and hole near the band edges and a deep electron form the valence band. Since this process results in a trapped electron, it will eventually lead to NR recombination. Thus, energy transfer and Auger trapping have the identical dependencies on the charge carrier concentrations and can be both included to the SRH theory as a third order trapping term:

$$k_E np(N - n_t) \quad (10.2)$$

Finally, the kinetic equations of the SRH+ model can be written as:

$$\frac{d}{dt}n(t) = G(t) - k_r np - k_t n(N - n_t) - k_E np(N - n_t) - k_A np^2 \quad (10.3)$$

$$\frac{d}{dt}n_t(t) = k_t n(N - n_t) + k_E np(N - n_t) - k_n n_t p \qquad (10.4)$$

$$\frac{d}{dt}p(t) = G(t) - k_r np - k_n n_t p - k_A np^2 \qquad (10.5)$$

To summarize, the effects on charge dynamics related to photon recycling in the broad meaning of this term (processes in both far and near field) are included in to the SHR+ model employed herein by adjusting the coefficients k_r and k_E.

Supplementary Note 11. CW regimes for different models (data for Figure 4 in the main text)

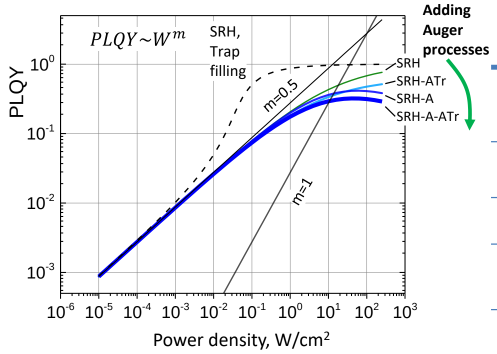

For all models:

$k_t N = 5 \times 10^6$ s^{-1} $\quad$ $k_r = 2.8 \times 10^{-12}$ cm^{3}s^{-1} $\quad$ $k_n = 1 \times 10^{-12}$ cm^{3}s^{-1}

SRH: $N = 1 \times 10^{20}$ cm^{-3}

SRH - Trap filling: $N = 1 \times 10^{17}$ cm^{-3}

SRH-Tr: $N = 1 \times 10^{20}$ cm^{-3} $\quad$ $k_E = 5 \times 10^{-33}$ cm^{6}s^{-1}

SRH-A: $N = 1 \times 10^{20}$ cm^{-3} $\quad$ $k_A = 8 \times 10^{-31}$ cm^{6}s^{-1}

SRH-A-Tr: $N = 1 \times 10^{20}$ cm^{-3} $\quad$ $k_E = 5 \times 10^{-33}$ cm^{6}s^{-1} $\quad$ $k_A = 8 \times 10^{-31}$ cm^{6}s^{-1}

Supplementary Figure 11.1. Data for Figure 4 c.

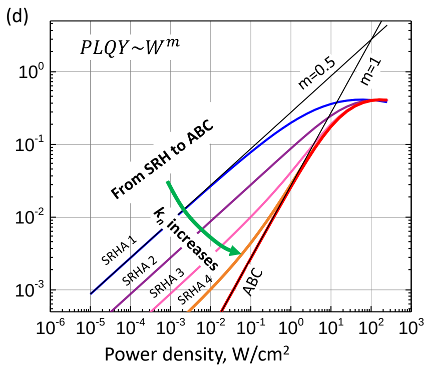

For all SRHA models:

$k_r = 2.8 \times 10^{-12}$ cm^{3}s^{-1} $\quad$ $k_A = 8 \times 10^{-31}$ cm^{6}s^{-1}

$N = 1 \times 10^{20}$ cm^{-3} $\quad$ $k_t N = 5 \times 10^6$ s^{-1}

SRHA 1: $k_n = 1 \times 10^{-12}$ cm^{3}s^{-1}

SRHA 2: $k_n = 1 \times 10^{-11}$ cm^{3}s^{-1}

SRHA 3: $k_n = 1 \times 10^{-10}$ cm^{3}s^{-1}

SRHA 4: $k_n = 1 \times 10^{-9}$ cm^{3}s^{-1}

For the ABC model:

$A = k_t N = 5 \times 10^6$ s^{-1} $\quad$ $B = k_r = 2.8 \times 10^{-12}$ cm^{3}s^{-1} $\quad$ $C = k_A = 8 \times 10^{-31}$ cm^{6}s^{-1}

Supplementary Figure 11.2. Data for Figure 4 d.

PLQY(W) dependences for the SRH and the SRH+ models are obtained by numerically solving Eqs (9.32-9.36). PLQY(W) dependence for the ABC model is obtained by numerically solving Eqs.(9.10-9.11). All these equations are solved using MATLAB.

Supplementary Note 12. Fitting procedures for all models

The fitting procedure for the ABC model

1. From the PLQY values at low excitation fluence (P3, P4) in the single pulse regime (Supplementary Figure 12.2) we estimate $\frac{k_r}{A}$ value using Eq. (9.17).

2. Repetition rate dependence of PLQY at high pulse fluence (P5) is fitted by the theoretical dependence obtained numerically by solving Eqs. (9.7-9.8). The fitting parameters are A, B and C.

The fitting procedure for SRH+ model

1. We estimate $k_t N$ product value by fitting the decay curves at low intensities with an exponential function using Eqs. (9.47) and (9.58). See Supplementary Figure 12.1.

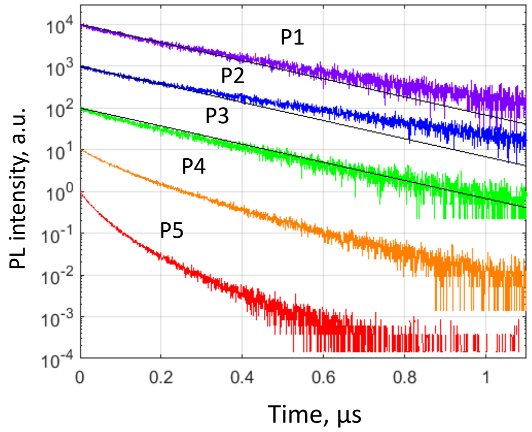

Supplementary Figure 12.1. PL Decay curves for the PMMA coated MAPI film (G/P/MAPI/P) at 100 KHz repetition rate for different pulse fluences. Black lines represent the exponential dependence $\exp(-k_t N t)$. The PL intensity is scaled individually for each pulse fluence to display all decays in one figure.

2. From the PLQY values at low excitation fluencies (P3, P4) in the single pulse regime (Supplementary Figure 12.2) we estimate $\frac{k_r}{k_t N}$ value using Eq. (9.48).
3. Power density dependence of PLQY in Quasi-CW regime is fitted by the theoretical dependence obtained numerically by solving Eqs. (9.32-9.36). The fitting parameters are N, k_n, k_A, and k_E.

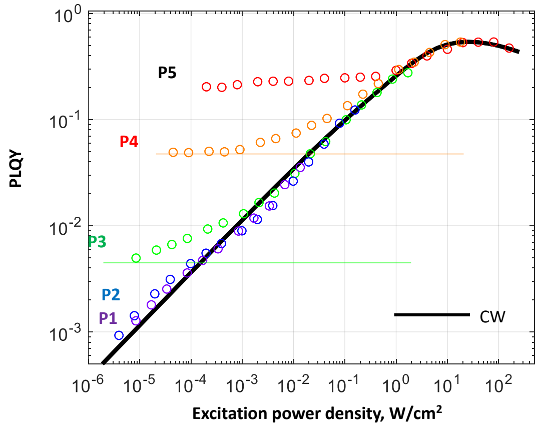

Supplementary Figure 12.2. Power density dependence of PLQY at different pulse energies. Straight horizontal lines show the theoretical estimation of PLQY in the single pulse regime (from Eq. (9.48)) for P2 and P3 pulse fluences. Thick black line shows the obtained theoretical PLQY dependence in the quasi-CW regime Eqs. (9.32-9.36). The sample is the G/P/MAPI/P.

Block schemes of the fitting algorithms for the ABC and SRH+ models.

1) SRH+

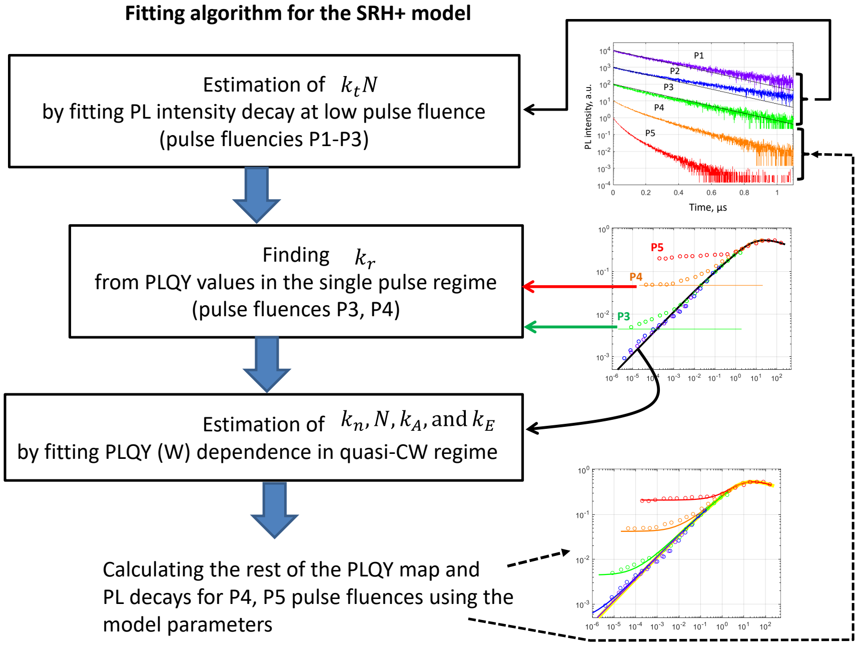

2) ABC

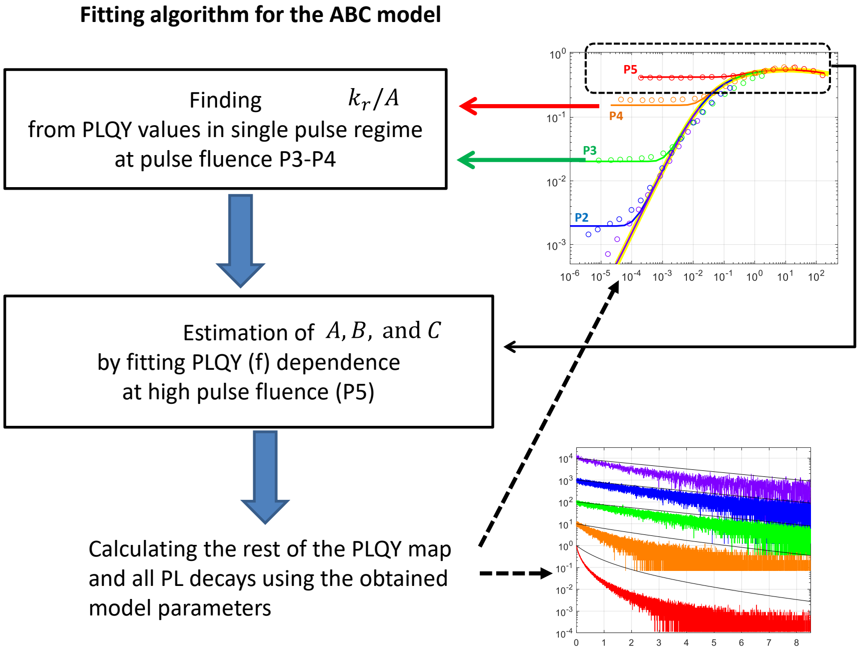

3) SRH. To plot the results of the SRH model we use the parameters obtained for the SRH+ model. In other words, the fit by the SRH model is obtained using the best fit parameters from the SRH+ with k_A and k_E set to zero. That is why the predictions of the SRH and SRH+ are identical at the low power regime.

Supplementary Note 13. Results of the fitting of the experimental data by ABC, SRH and SRH+ models

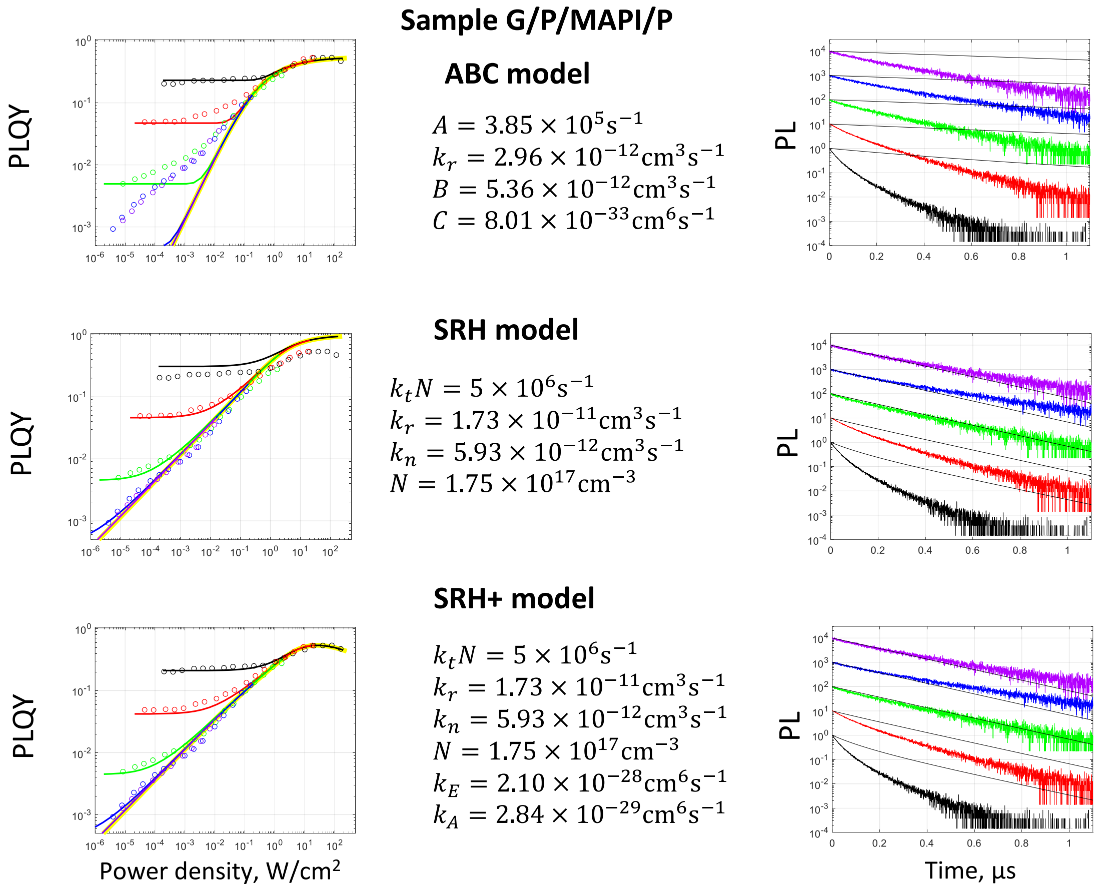

Supplementary Figure 13.1. Fitting results the PLQY maps and PL decays by all models for G/P/MAPI/P sample.

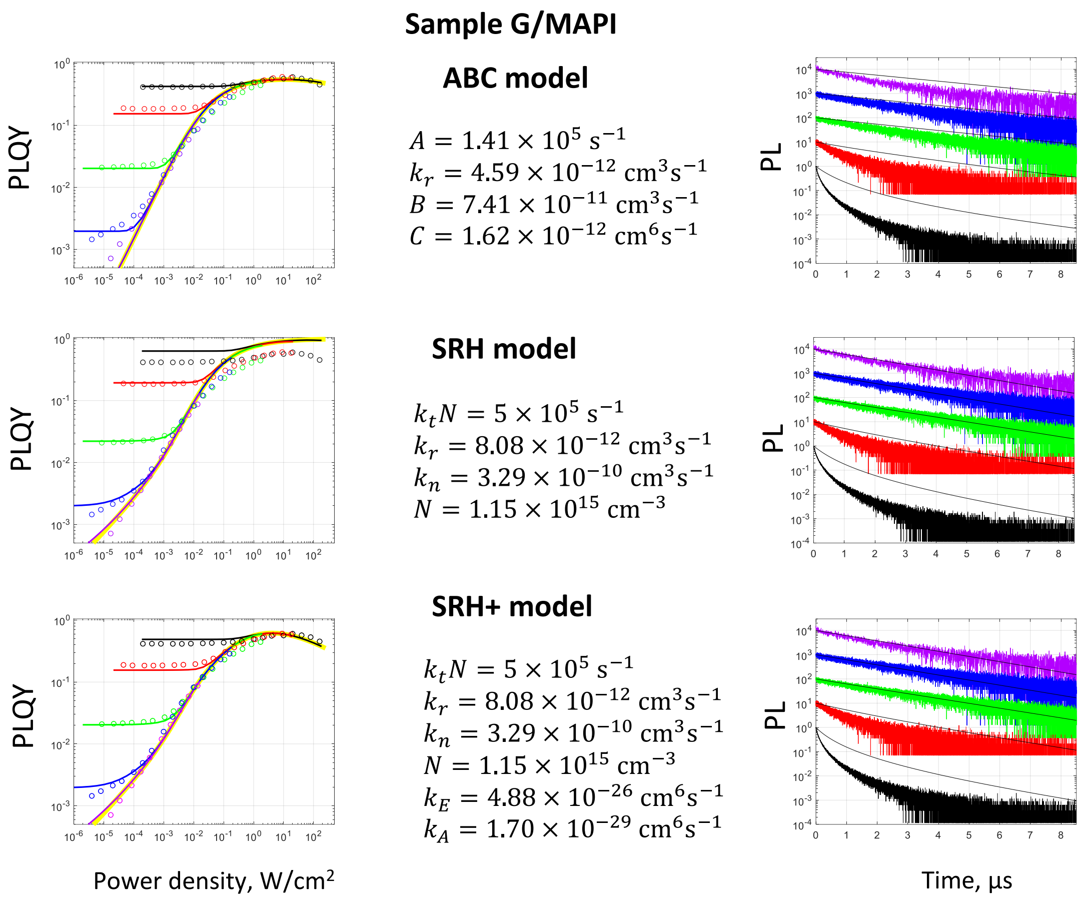

Supplementary Figure 13.2. Fitting results the PLQY maps and PL decays by all models for G/MAPI sample. Lines are theoretical curves. Note the complete failure of the ABC model to explain PL decays, although the PLQY maps is fitted reasonably well.

PLQY(f,P) and *PL(t)* dependences for the SRH and SRH+ models are obtained by numerically solving the kinetic equations Eqs. (9.18-9.20). *PLQY(f,P)* and *PL(t)* dependences for the ABC model are obtained by numerically solving the kinetic equation Eq.(9.7). Corresponding kinetic equations are solved for many periods of excitation until periodic solutions are reached. Numerical simulations are conducted using MATLAB.

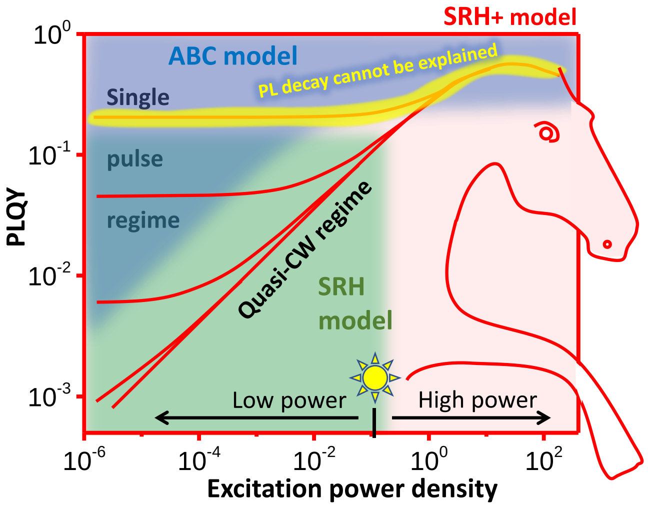

Supplementary Figure 13.3. The cartoon shows the ability of the ABC (blue coloration), SRH (green coloration) and SRH+ (red) models to fit the PLQY(f,P) map for $MAPbI_3$ perovskite. The high pulse power region where PL decay cannot be explained by the models is highlighted by yellow. Resembling of the PLQY map to a “running horse with a mane” is illustrated.

Supplementary references:

1. An, Q. *et al.* Enhancing the Open-Circuit Voltage of Perovskite Solar Cells by up to 120 mV Using π-Extended Phosphoniumfluorene Electrolytes as Hole Blocking Layers. *Adv. Energy Mater.* **9**, 1901257 (2019).

2. Butscher, J. F. *et al.* Enhancing the Open-Circuit Voltage of Perovskite Solar Cells by Embedding Molecular Dipoles within Their Hole-Blocking Layer. *ACS Appl. Mater. Interfaces* **12**, 3572–3579 (2020).

3. Tian, Y. & Scheblykin, I. G. Artifacts in Absorption Measurements of Organometal Halide Perovskite Materials: What Are the Real Spectra? *J. Phys. Chem. Lett.* **6**, 3466–3470 (2015).

4. Kudriashova, L. G. *et al.* Impact of interfaces and laser repetition rate on photocarrier dynamics in lead halide perovskites. *J. Phys. Chem. Lett.* **8**, 4697–4703 (2017).

5. Chirvony, V. S. *et al.* Interpretation of the photoluminescence decay kinetics in metal halide perovskite nanocrystals and thin polycrystalline films. *J. Lumin.* **221**, 117092 (2020).

6. Herz, L. M. Charge-Carrier Mobilities in Metal Halide Perovskites: Fundamental Mechanisms and Limits. *ACS Energy Lett.* **2**, 1539–1548 (2017).

7. Richter, J. M. *et al.* Enhancing photoluminescence yields in lead halide perovskites by photon recycling and light out-coupling. *Nat. Commun.* **7**, 13941 (2016).

8. Kühn, S., Mori, G., Agio, M. & Sandoghdar, V. Modification of single molecule fluorescence close to a nanostructure: Radiation pattern, spontaneous emission and quenching. *Mol. Phys.* **106**, 893–908 (2008).

9. Abakumov, V. N., Perel, V. I. & Yassievich, I. N. *Nonradiative Recombination in Semiconductors*. (North-Holland, Amsterdam, 1991).
